# Ultrasound-guided versus stereotactically navigated ventriculoperitoneal shunt placement: a randomized clinical trial

**DOI:** 10.1186/s12987-026-00833-2

**Published:** 2026-06-26

**Authors:** Severina Leu, Tim Hallenberger, Jonathan Rychen, Lea Pacan, Evangelia Christodoulou, Kristine Blackham, Ladina Greuter, Florian Samuel Halbeisen, Ethan Taub, Birgit Westermann, Raphael Guzman, Luigi Mariani, Jehuda Soleman

**Affiliations:** 1 Department of Neurosurgery, University Hospital of Basel, Spitalstrasse 21, Basel, 4031 Switzerland; 2 Department of Neuroradiology, University Hospital of Basel, Basel, Switzerland; 3 Surgical Outcome Research Center Basel, University Hospital of Basel, Basel, Switzerland; 4 Faculty of Medicine, University of Basel, Basel, Switzerland

**Keywords:** Ultrasound navigation, Stereotactic navigation, Ventriculoperitoneal shunt

## Abstract

**Background:**

Accurate catheter positioning is essential for optimal outcomes in ventriculoperitoneal shunt (VPS) surgery, while shorter operative times lower infection risk and costs. Navigated VPS placement, using either intraoperative ultrasound-guided (US-G) or stereotactically guided (ST-G) navigation, enhances catheter accuracy and reduces revision rates. However, high-quality studies comparing the two navigation methods are lacking. We aim to compare surgical intervention time, accuracy, and safety of US-G to ST-G VPS placement.

**Methods:**

The Navigated VPS (NAVPS) trial was an investigator-initiated, randomized trial conducted from February 2020 to June 2024 in the Neurosurgical Department of the University Hospital of Basel. Consecutive adults undergoing VPS placement were included. Out of 153 screened participants, 134 participants were included. Participants were randomized 1:1 to receive either US-G or ST-G insertion of the ventricular catheter. The primary outcome was surgical intervention time. An intention to treat analysis was performed calculating surgical intervention time differences. Secondary outcomes were accuracy of catheter positioning, number of ventricular puncture attempts, and VPS dysfunction and complication rates. The study follow-up lasted 6 months.

**Results:**

Of 134 participants, 66 were assigned to US-G and 68 to ST-G. The mean (SD) age was 73 (55.3 to 78) and 66 (54.5 to 73) years for the US-G and ST-G, respectively, and 58 participants (45.7%) were female. The US-G group had significantly shorter surgical intervention times compared to the ST-G group (-11.5 min; 95% CI -18.5 to -4.5; *P* = 0.002). The number of ventricular puncture attempts was significantly higher in the US-G group, while accuracy of catheter placement, and VPS dysfunction and complications rates were comparable in both groups.

**Conclusions:**

The NAVPS trial shows US-G VPS placement to be more time-efficient, while accuracy of catheter placement and complication rates seem to be comparable to ST-G placement. US-G can be efficiently and safely used in clinical practice.

**Trial registration:**

clinicalTrials.gov Identifier: NCT04450797, date of registration: 22.06.2020.

**Supplementary Information:**

The online version contains supplementary material available at 10.1186/s12987-026-00833-2.

## Background

 Ventriculoperitoneal shunt (VPS) placement is among the most common neurosurgical procedures, indicated for conditions ranging from (NPH) pressure hydrocephalus in elderly patients to VPS-dependency after subarachnoid haemorrhage, infection, or trauma in patients of all ages [1–3]. Short operative times are essential for both cost-effectiveness and patient safety, since longer duration of surgery might lead to higher complication rates, particularly infections [4–6].

The position of the proximal ventricular catheter is a key determinant of shunt function [7–10]. Traditionally, VPS insertion has been performed freehand, yet this approach carries a substantial risk of catheter malposition [11]. Optical stereotactic navigation enables highly accurate catheter placement but requires head fixation and time-consuming preoperative setup [12–14]. Electromagnetic navigation eliminates the need for head fixation [15, 16], but may offer lower accuracy and still requires preoperative planning and registration [17–21].

Ultrasound-guided (US-G) VPS placement has emerged as an appealing alternative, offering real-time visualization without the need for preoperative imaging, head fixation, or registration [22, 23]. Studies demonstrate that US-G catheter placement is more accurate than freehand placement, and intraoperative guidance reduces shunt failure and revision rates [24–26]. A retrospective cohort study found comparable accuracy between stereotactically-guided (ST-G) and US-G placement, both outperforming freehand placement [24].

Navigation methods that are both precise and practical have the potential to become the standard of care in VPS surgery, provided they do not prolong operative time or increase costs. However, most available evidence stems from small retrospective series [15, 23–26], and prospective randomized trials directly comparing US-G and ST-G VPS placement remain lacking.

To address this gap the Navigated VPS (NAVPS) randomized clinical trial was conducted. The trial aimed to assess the difference in surgical intervention time between US-G and ST-G VPS placement.

## Methods

### Trial design and oversight

This trial was overseen by the Department of Neurosurgery at the University Hospital of Basel and was funded by the Gottfried and Julia Bangerter-Rhyner Foundation and by the Research Foundation of the University Hospital of Basel. The trial protocol was published previously [27], is available on Zenodo (www.zenodo.org, doi: 10.5281/zenodo.17378539) and is available as Additional File 1. The trial protocol was approved by the local ethics committee (EKNZ 2019–02157).

Data analysis was performed by a trial statistician (FS. H.) who attests to the integrity of the analysis and the accuracy and completeness of the reported data. The centre and all investigators guarantee the accuracy and completeness of the data, adherence to the trial protocol, and precise reporting of adverse events. There was no industry involvement in the trial.

### Participants

Eligible participants were adults (≥ 18 years) scheduled for VPS placement. Exclusion criteria were age < 18 years, lack of consent, emergency surgery without time for navigation setup, revision without full shunt replacement, and ventriculoatrial or ventriculopleural shunt placement. Detailed eligibility criteria are provided in the trial protocol [27] (10.5281/zenodo.17378539, Additional File 1).

### Trial treatment

Participants were randomized in a 1:1 ratio to US-G (intervention) or ST-G (control) VPS insertion using REDCap [28]. The participants were additionally stratified by age (< 40 or ≥ 40 years) to control for aetiological differences of hydrocephalus. Participants were blinded to their assigned navigation method, while blinding of the surgeons was not possible. The primary outcome was recorded by a blinded assessor, while radiological outcomes were assessed by two independent and blinded neuroradiologists.

**Fig. 1:**
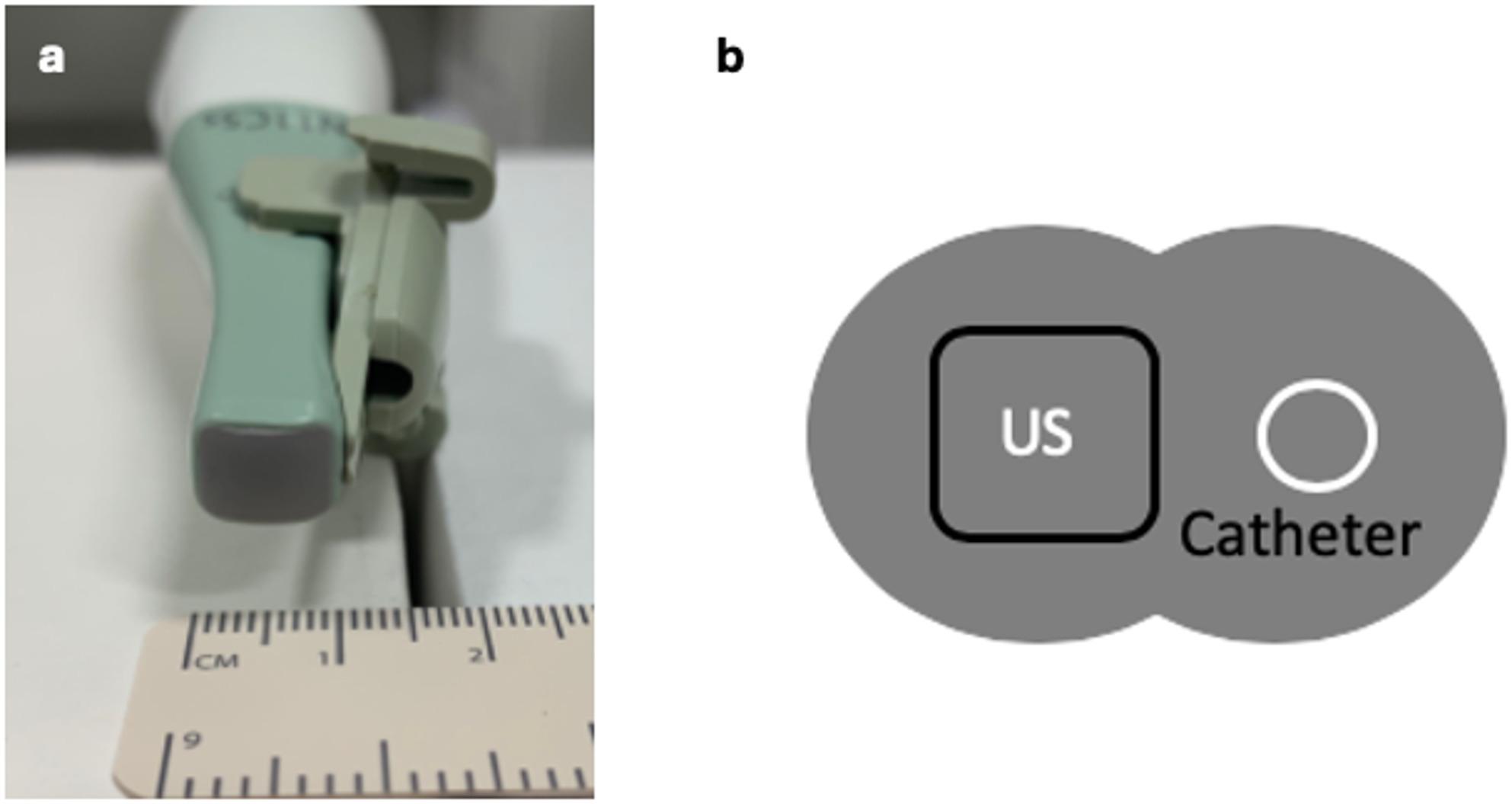
(**a**) Ultrasound burr-hole probe with attached trajectory guide, (**b**) Double burr-hole access enables the ventricular catheter to be passed adjacent to the burr-hole probe

US-G was performed using BK Medical US (BK Medical Medizinische Systeme GmbH, Quickborn, Germany) burr hole probe (9063 N11C5S, 11 − 5 MHz) with an attached trajectory guide (Fig. 1a). The head was supported in a horseshoe head holder. Following skin incision at Kocher’s point (11 cm from the Nasion and 3 cm lateral to the midline), two overlapping burr holes were placed (Fig. 1b), and the dura was opened in a cruciate fashion.

**Fig. 2:**
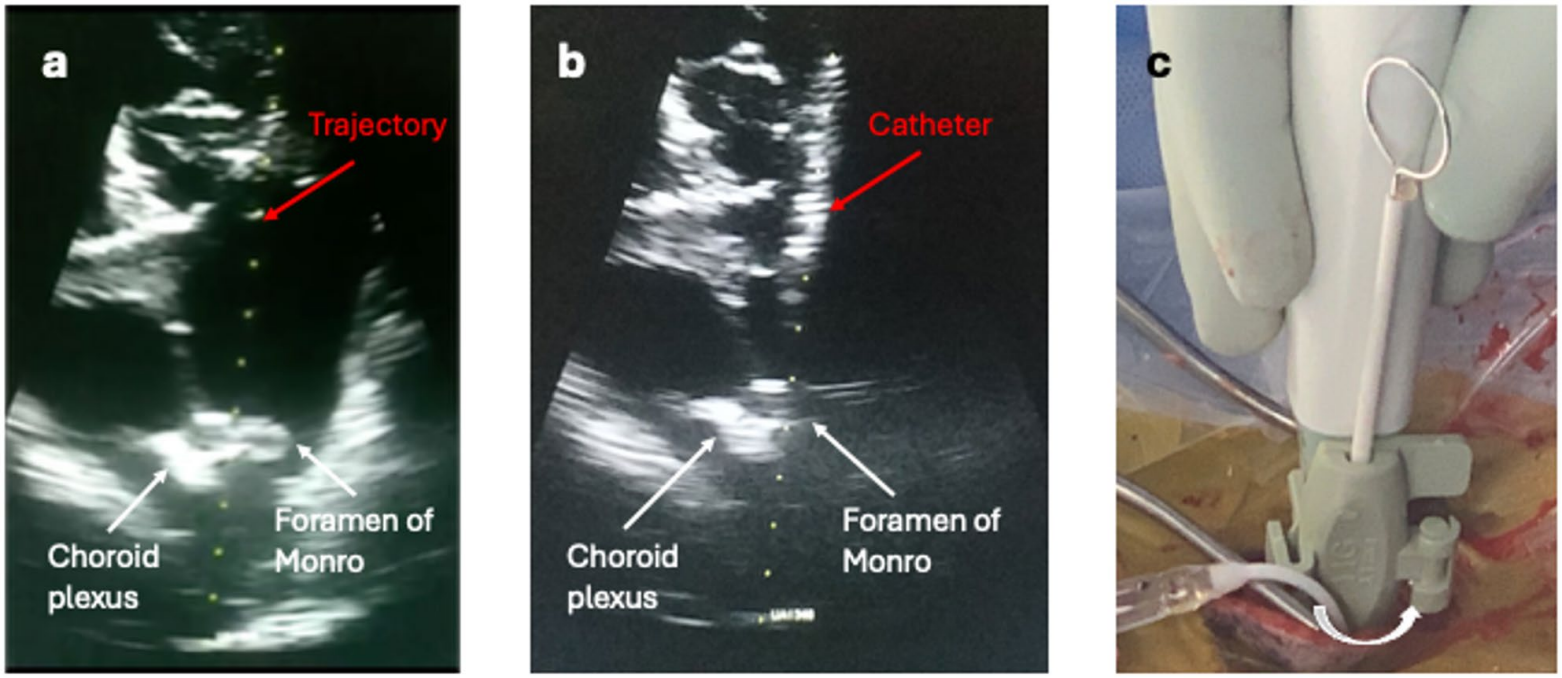
Details of the ultrasound navigation technique (**a**) Visualisation of the lateral ventricles, the choroid plexus, and the foramina of Monro, (**b**) Insertion of the ventricular catheter under real-time ultrasonic guidance along the trajectory towards the foramen of Monro, (**c**) Verification of cerebrospinal fluid (CSF) flow, opening of the trajectory guide and removal of the US burr-hole probe while the catheter is held in place. Note: Curved white arrow illustrates opening of the trajectory guide

Using the US burr-hole probe, the lateral ventricles, choroid plexus, and foramina of Monro were identified (Fig. 2a). An antibiotic-impregnated ventricular catheter was then advanced through the trajectory guide under real-time ultrasound into the ventricle at the level of the foramen of Monro (Fig. 2b). After catheter placement, the US trajectory guide was opened and the US probe removed while maintaining catheter position (Fig. 2c).

After confirmation of CSF flow, the catheter was trimmed and connected to a burr-hole reservoir (CODMAN^®^ HOLTER^®^ RICKHAM^®^ reservoir), a programmable valve, and peritoneal catheter. After subcutaneous tunneling, the distal peritoneal catheter was inserted laparoscopically into the peritoneum using the Seldinger technique [29–31].

**Fig. 3:**
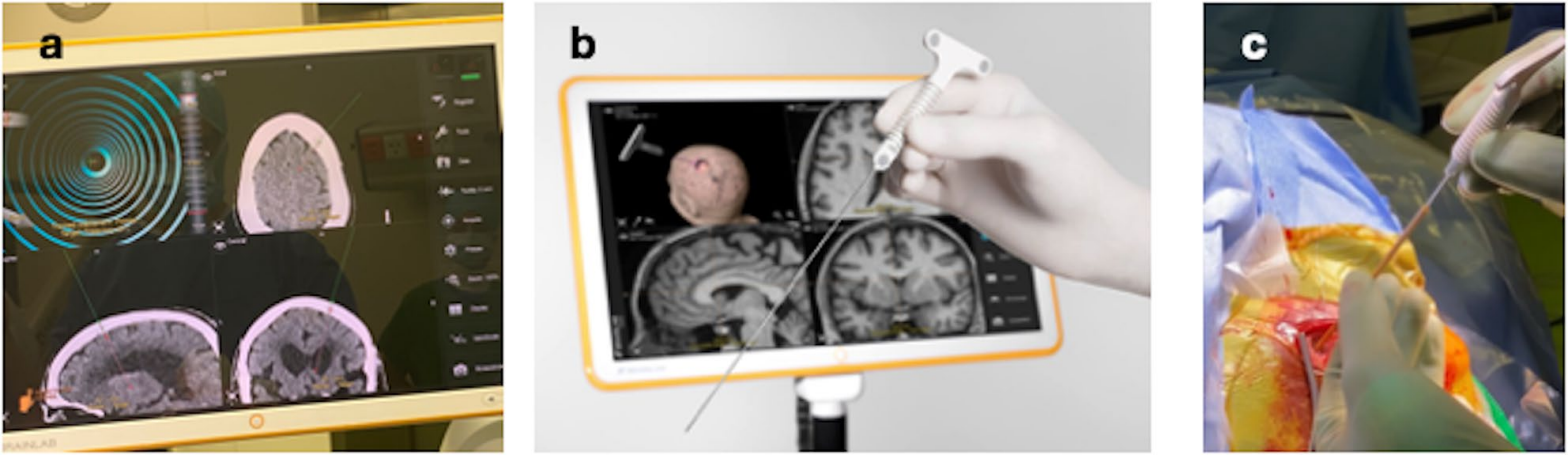
Details of the stereotactic navigation technique (**a**) Preoperative trajectory planning using Brainlab software and workstation, (**b**) and (**c**) Ventricular catheter insertion using the navigated disposable stylet

ST-G was performed with the patient’s head secured in a skull clamp (DORO^®^ QR3 Skull Clamp, Black Forest Medical, Freiburg, Germany). Preoperative trajectory planning was conducted using the Brainlab workstation and software (version 3.1, Brainlab AG, Munich, Germany, Fig. 3a), and catheter insertion was performed with the Brainlab Navigated Disposable stylet (Fig. 3b and c).

Connection to the distal shunt components (burr-hole reservoir, programmable valve, and peritoneal catheter), subcutaneous tunnelling, and intraperitoneal insertion were performed as described for the US-G cohort.

Prophylactic anticonvulsant medication was not used, but all participants received a single dose of antibiotics 30 min before the skin incision. In 27 (21.3%) cases the burr hole and shunt insertion was done on the left side, while we only included patients with frontal VPS placement.

### Outcomes

The primary outcome was surgical intervention time, defined as the total time the neurosurgeon spends in the operating room (OR), from the first patient intervention (e.g., positioning, head clamping) to the completion of the neurosurgical part of the procedure. In the ST-G group, 5 min were added for preplanning of the trajectory on the Brainlab Workstation (Additional File 2). The general surgery part of the procedure (laparoscopically assisted peritoneal catheter insertion) was excluded to minimize bias.

Secondary outcomes were operation time and anaesthesia time in minutes, number of ventricular puncture attempts, catheter placement [according to Yim et al. into grades I to IV (grade I: catheter terminates in the ipsilateral frontal horn, grade II: catheter terminates in the contralateral frontal horn, grade III: catheter terminates in non-targeted CSF spaces, grade IV: catheter terminates in the brain parenchyma [9])], ventricle volumes (quantified using the AI-based TotalSegmentator tool [32, 33]) pre- and postoperatively in cm^3^ (number and relative change), Evans Index (measured as the ratio of the maximum frontal horn diameter to the maximum internal skull diameter on the same axial CT plane [34]) pre- and postoperatively (number and relative change), complications (infection, bleeding, complications associated with navigation method), revision surgery and reason for revision. The study included six visits as described in Additional File 3.

### Statistical analysis

Assuming an intervention time of 63 min ± 28.7 min for the US-G arm, the sample size was set to identify a time difference of 15 min between the study arms at a two-sided significance level of 5%, allowing for 1% drop-out and 5% surgeon non-adherence, 130 participants were required (Additional File 4). Due to a higher-than-expected dropout rate observed after randomizing 130 participants, the ethics committee approved the inclusion of additional participants, raising the final number of participants randomized to 134. All enrolled participants were analysed according to their assigned treatment regardless of loss to follow-up or protocol violations, such as incomplete adherence to medication.

The difference in surgical intervention time was analysed by linear regression including a crude model (group only) and an adjusted model (groups corrected for body mass index (BMI), previous burr hole, surgeon experience, and underlying disease). As a secondary analysis operation and anaesthesia, catheter position, shunt dysfunction, complications, and revision surgery time were analysed using logistic regression. Catheter position grading, ventricle/Evans Index and improvement were analysed with ordinal logistic regression. Puncture attempts were analysed using Poisson regression, while absolute/relative changes in ventricle volumes and Evans Index were analysed using linear regression.

No per-protocol analysis was undertaken, as all participants received treatment as randomized and the intention-to-treat and per-protocol populations were therefore identical. No subgroup analyses were prespecified or performed. Sensitivity analyses were conducted to assess the robustness of the primary and secondary outcomes. For the primary outcome, median regression, robust regression, and multiple imputation of missing data were performed, yielding results consistent with the main analysis. For operation time and anaesthesia time, robust regression was applied, and for the number of puncture attempts, a quasi-Poisson model was used to account for overdispersion; all produced similar conclusions to the primary analysis.

Full details of the statistical analysis are provided in the trial protocol [27] (www.zenodo.org, 10.5281/zenodo.17378539) that is available together with the Statistical Analysis Plan as Additional File 1.

## Results

**Fig. 4:**
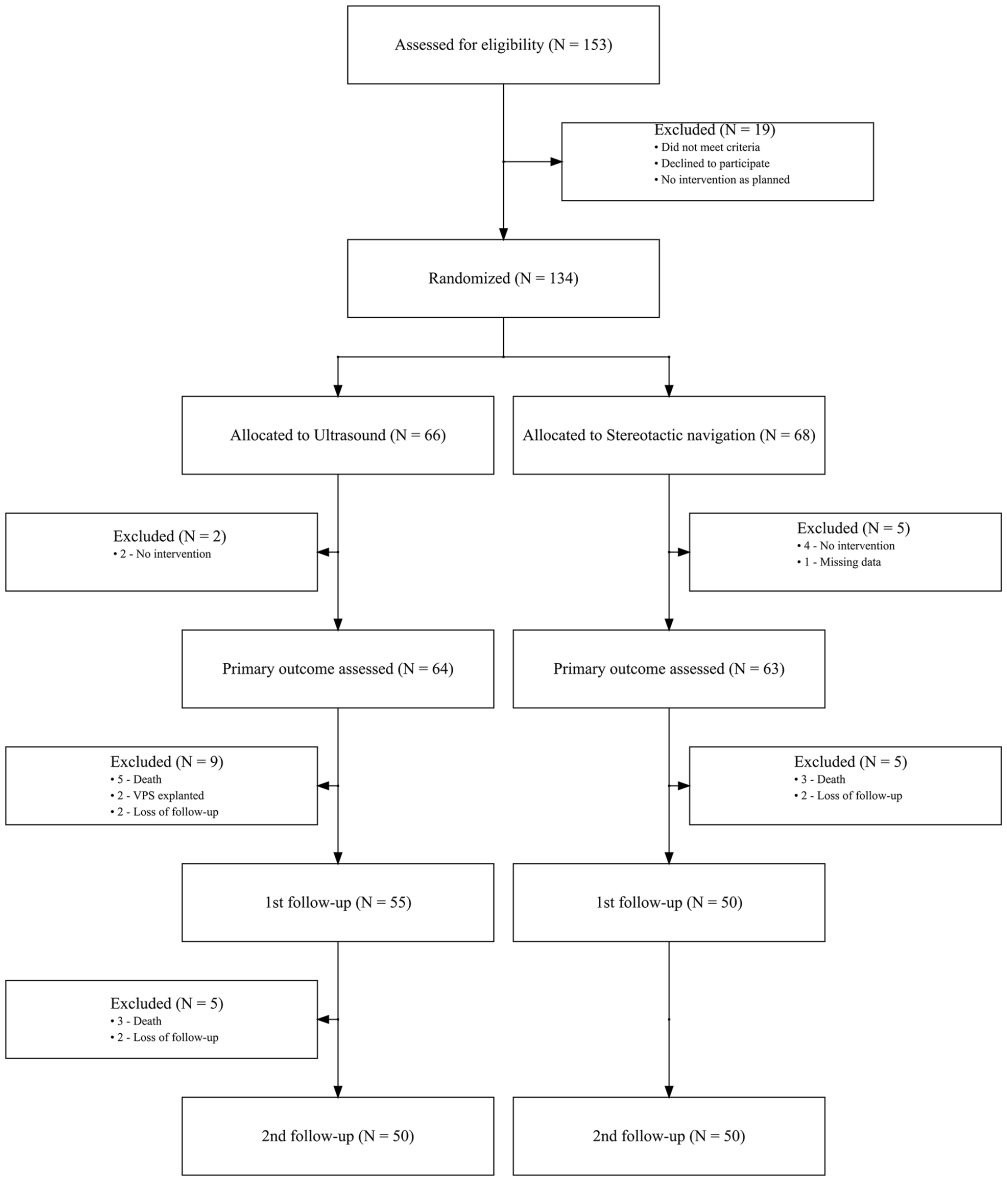
Consort flow diagram

A total of 153 participants were screened, and 134 participants were enrolled at the University Hospital of Basel between February 2020 and June 2024. The distribution between trial groups is shown in Fig. 4. Sixty-six participants were randomized to the US-G and 68 to the ST-G group. At the 6-month follow-up, 4 participants in the US-G and 2 in the ST-G group were lost to follow-up. Additionally, 8 participants in the US-G and 3 in the ST-G group died during the trial period, while 2 participants in the US-G group had their VPS explanted during follow-up (Fig. 4).

Baseline demographic and clinical characteristics were similar in both treatment groups **(**Table 1**).** Median (IQR) age was 73 years (55.3–78) in the US-G and 66 years (54.5–73) in the ST-G group. Fifty-eight participants (45.7%) were female. Normal pressure hydrocephalus was the most common aetiology (46.5%), followed by subarachnoid haemorrhage (18.1%), other bleeding (11.8%), and tumor (11.8%), while 36.2% of participants had a prior EVD and 5.5% a prior VPS surgery. At admission, gait disturbance (78%), urinary incontinence (44.1%), and dementia (39.4%) were the most common symptoms. Median GCS at admission was 15 in both groups (Table 1).

**Table 1:** Baseline characteristics of the treated patient population

	Total (*N* = 127)	US-G navigation (*N* = 64)	ST-G navigation (*N* = 63)
Age (Median & IQR)	69 (54.5 to 75)	73 (55.25 to 78)	66 (54.5 to 73)
Sex			
Female	58 (45.67)	23 (35.94)	35 (55.56)
Male	69 (54.33)	41 (64.06)	28 (44.44)
Height (Median & IQR)	169 (164 to 178)	170 (165 to 178.5)	168 (162.5 to 175)
BMI (Median & IQR)	25.39 (23.07 to 29.14)	25.14 (23.17 to 27.84)	26.57 (22.93 to 30.55)
Admission Evens Index (Median & IQR)	0.38 (0.33 to 0.42)	0.38 (0.34 to 0.42)	0.38 (0.33 to 0.42)
Admission Volumetry of side ventricles (in cm3) (Median & IQR)	125 (81 to 158.5)	119.5 (90.25 to 157.25)	130 (78 to 165.5)
Admission GSC (Median & IQR)	15 (14 to 15)	15 (14 to 15)	15 (12 to 15)
Admission Modified Rankin Scale			
0	6 (4.72)	4 (6.25)	2 (3.17)
1	21 (16.54)	11 (17.19)	10 (15.87)
2	34 (26.77)	19 (29.69)	15 (23.81)
3	15 (11.81)	10 (15.62)	5 (7.94)
4	28 (22.05)	14 (21.88)	14 (22.22)
5	23 (18.11)	6 (9.38)	17 (26.98)
Admission Glasgow Outcome scale			
5	42 (33.07)	25 (39.06)	17 (26.98)
4	39 (30.71)	20 (31.25)	19 (30.16)
3	38 (29.92)	19 (29.69)	19 (30.16)
2	8 (6.3)	0 (0)	8 (12.7)
1	0 (0)	0 (0)	0 (0)
Prior VP shunt OP			
Yes	7 (5.51)	5 (7.81)	2 (3.17)
No	120 (94.49)	59 (92.19)	61 (96.83)
Prior EVD OP			
Yes	46 (36.22)	20 (31.25)	26 (41.27)
No	81 (63.78)	44 (68.75)	37 (58.73)
Prior head operation			
Yes	37 (29.13)	15 (23.44)	22 (34.92)
No	90 (70.87)	49 (76.56)	41 (65.08)
Underlying disease causing hydrocephalus			
Normal pressure hydrocephalus	59 (46.46)	34 (53.12)	25 (39.68)
Subarachnoid hemorrhage	23 (18.1)	9 (14.1)	14 (22.22)
Other bleeding	15 (11.8)	8 (12.5)	7 (11.11)
Tumor	15 (11.8)	8 (12.5)	7 (11.11)
Trauma	8 (6.3)	2 (3.12)	6 (9.52)
Other	7 (5.51)	3 (4.69)	4 (6.35)
Admission Headache	42 (33.07)	22 (34.38)	20 (31.75)
Admission Vomitus	18 (14.17)	6 (9.38)	12 (19.05)
Admission Coma	10 (7.87)	2 (3.12)	8 (12.7)
Admission Gait disturbance	99 (77.95)	53 (82.81)	46 (73.02)
Admission Dementia	50 (39.37)	29 (45.31)	21 (33.33)

### Primary outcome

**Fig. 5:**
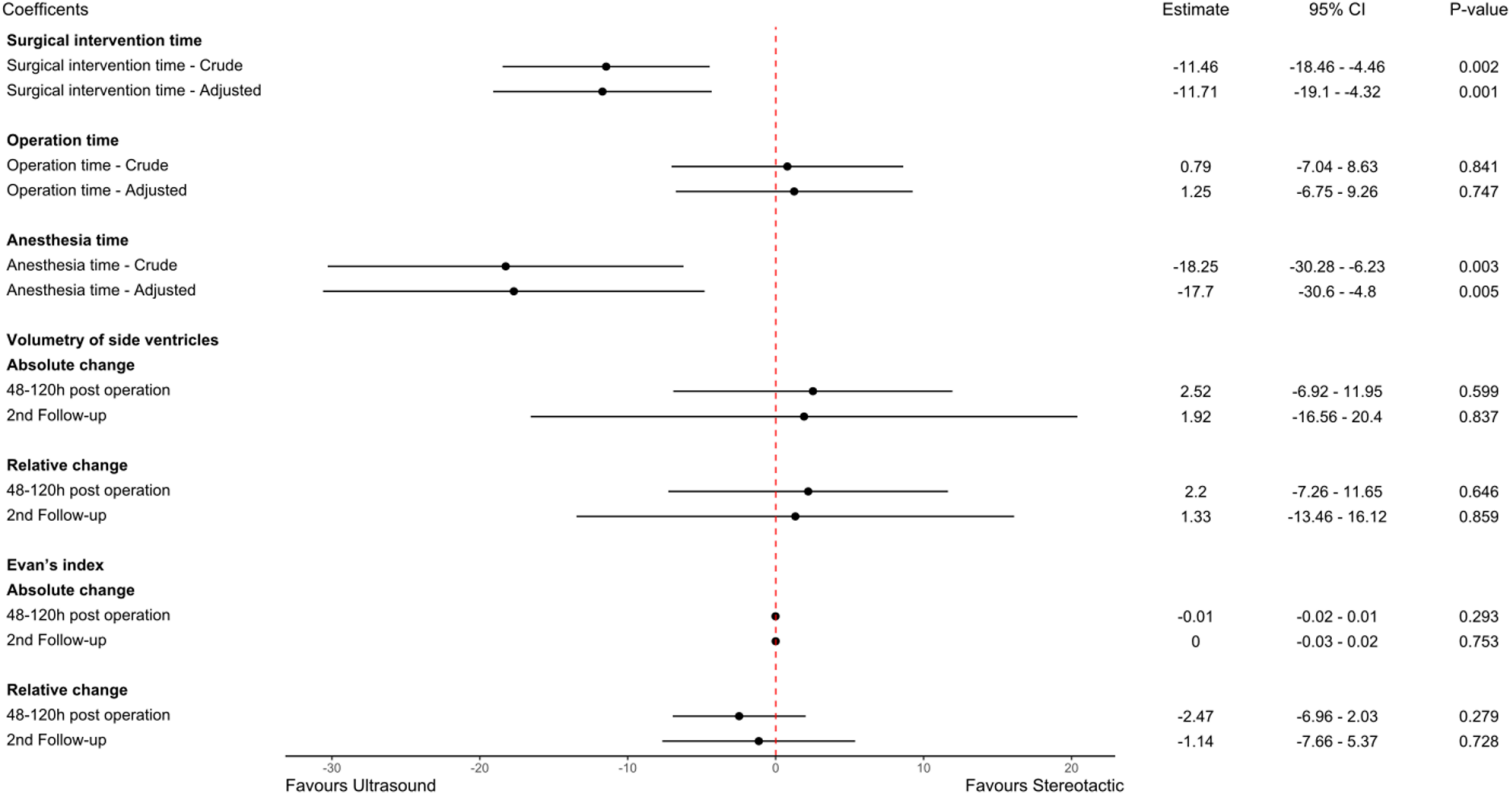
Forest plot depicting the impact of ultrasound guided (US-G) navigation versus stereotactically guided (ST-G) navigation on continuous study outcomes

US-G VPS placement reduced surgical intervention time by -11.5 min compared to ST-G (crude model: 95% CI -18.5 to -4.5, *p* = 0.002, adjusted model: -11.7 min, 95% CI -19.1 to -4.3, *p* = 0.001, Fig. 5).

Tumor aetiology was associated with longer surgical intervention times, while BMI, previous burr hole, surgeon experience, and other aetiologies showed no significant effect (Table 2; Fig. 5, Additional File 5).

**Table 2:** Primary outcome: Surgical intervention time (min) in ultrasound-guided (US-G) and stereotactically guided (ST-G) ventriculoperitoneal shunt (VPS) placements

	Total (*N* = 127)	US-G navigation (*N* = 64)	ST-G navigation (*N* = 63)
Surgical intervention time in min (Mean & SD)	99 (20.51)	93.23 (18.92)	104.68 (20.56)
Surgical intervention time in min (Median & IQR)	96 (86 to 115)	90.5 (85.25 to 103.75)	100 (92 to 121)
**Linear regression analysis (Surgical intervention time (min))**			
Coefficients	**Estimates**	**95% CI**	**P-Value**
Crude Model			
US-G (vs. ST-G)	-11.46	-18.46 - -4.458	0.002
Adjusted Model			
US-G (vs. ST-G)	-11.71	-19.1 - -4.325	0.001
BMI	-0.3835	-1.136–0.369	0.294
Previous burr hole	-6.254	-17.15–4.648	0.237
Experience surgeon (years)	0.03258	-0.3605–0.4257	0.864
Underlying disease causing hydrocephalus			0.303
Subarachnoid haemorrhage	3.954	-8.008–15.92	
Other bleeding	0.6596	-13.28–14.6	
Tumor	13.65	1.501–25.79	
Trauma	2.198	-13.31–17.7	
Other	7.124	-9.084–23.33	

### Secondary outcomes

**Fig. 6:**
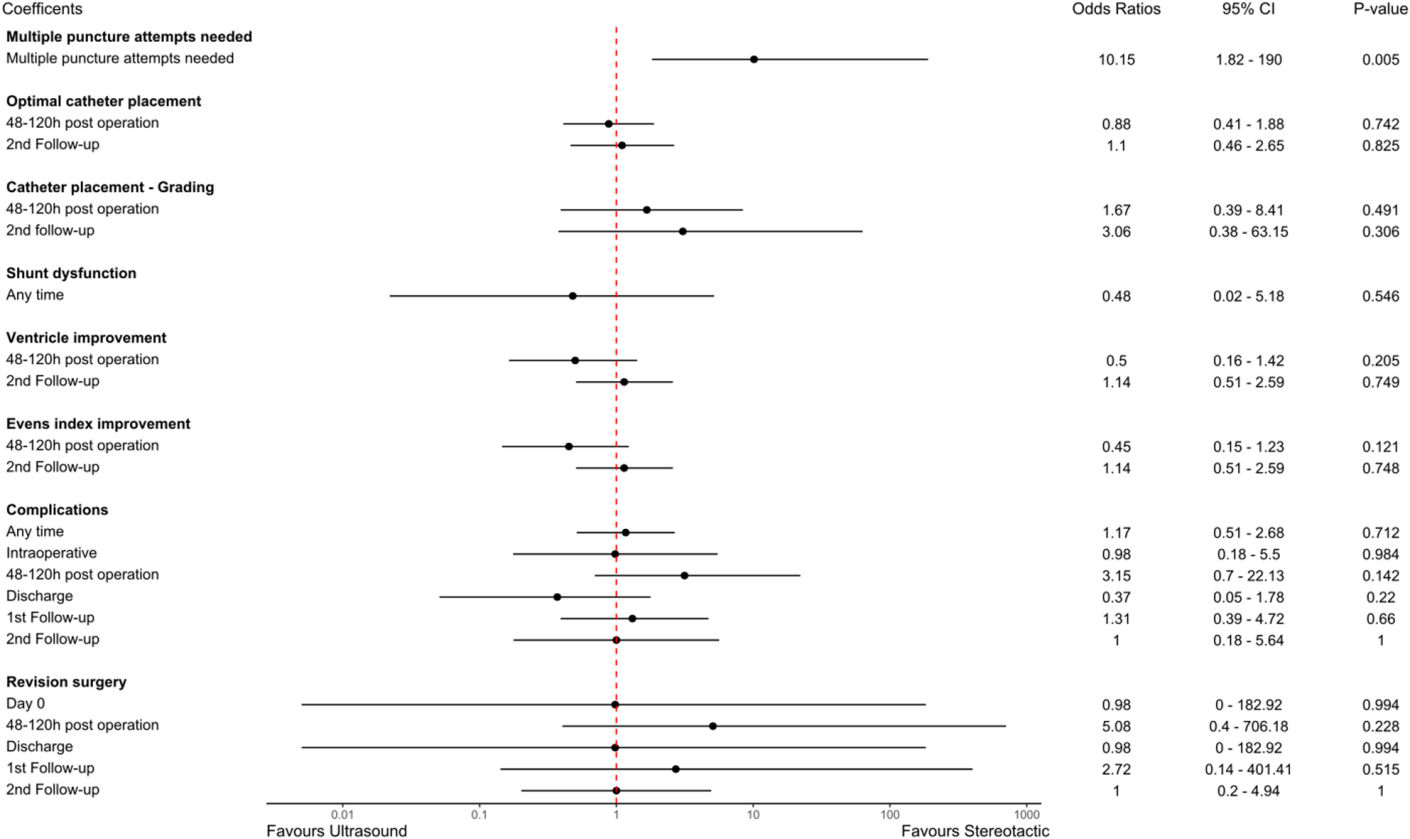
Forest plot depicting the impact of ultrasound guided (US-G) navigation versus stereotactically guided (ST-G) navigation on categorical study outcomes

No significant difference in operation time was observed between US-G and ST-G VPS placement (Additional Files 6 and 7). US-G was associated with significantly shorter anaesthesia time compared to ST-G (adjusted model: -17.7 min, 95% CI -30.6 to -4.8, *p* = 0.005). None of the covariates significantly affected anaesthesia time (Additional Files 8 and 9). US-G increased the number of puncture attempts, with a 15% higher rate (Rate Ratio: 1.2, 95% CI: 1.1 to 1.3, *p* = 0.004) and markedly higher odds for multiple attempts (OR: 10.2, 95% CI: 1.8 to 190, *p* = 0.005, Additional File 10). Catheter positioning, catheter position grading, and dysfunction rates did not differ significantly between groups (Additional Files 11–13). Fifty-nine participants in the US-G group (92.2%) and 60 participants in the ST-G group (95.2%) had grade I catheter positioning, with no grade IV placements and two grade III placements in both the US-G and ST-G groups (Additional File 12). Revision surgery occurred in 9 participants (7.1%), most commonly due to infection in 3 participants (2.4%). Only 2 revisions (1.6%) were due to proximal misplacement. Revision rates and infection-related revisions did not differ between groups (Additional File 14). Complication rates were similar between US-G and ST-G (Additional File 15). US -G was associated with improved functional outcomes at the second follow-up, but not at admission or earlier timepoints (Table 1 and Additional File 16). No significant differences were found in ventricle volume reduction or Evans Index improvement between the groups (Figs. 5 and 6, Additional Files 17 and 18).

## Discussion

This study compared surgical duration, accuracy, and safety between ultrasound-guided (US-G) and stereotactically guided (ST-G) ventriculoperitoneal shunt (VPS) placement. Total intervention time was significantly shorter with US-G navigation, primarily due to the absence of preoperative planning, head fixation, and registration. Operative time itself was similar between groups. Consequently, US-G navigation improves surgical efficiency, reducing anaesthetic exposure, perioperative risk, and recovery time. Although more ventricular puncture attempts were required with US-G navigation, most probably reflecting the technique’s learning curve, all repeated punctures followed identical trajectories, and no intraparenchymal haemorrhage occurred. In contrast, one minor, asymptomatic bleeding event was observed in the ST-G group. Overall complication rates, catheter placement accuracy, and shunt function outcomes were comparable, confirming that both navigation methods are equally safe and effective. These findings support US-G as a time-efficient and equally safe alternative to ST-G in VPS surgery.

Although prolonged surgical time has been associated with higher VPS complication rates, particularly infections [4–6], infection and overall complications were comparable between groups in our study. In the US-G cohort, a higher number of ventricular puncture attempts were required. This contrasts with findings by Unal and colleagues, who reported single-attempt catheter placement in all 18 US-G cases with normal-sized ventricles [35]. Accurate proximal catheter placement remains a key determinant of VPS function [7–10, 36]. Freehand insertion is associated with higher rates of malposition [7, 15, 24, 25]; in our previously published retrospective cohort 5.8% were classified as grade III – IV malpositions [11]. Consistent with prior studies showing that navigated placement improves accuracy and reduces revision rates [12, 13, 24–26], we found that both US-G and ST-G techniques achieve high accuracy [24]. In this trial, grade III malposition occurred in 3.1%, while no grade IV mispositioning were observed. Optimal placement was achieved in 70.1% of cases using navigation, compared with 49% in our historical freehand cohort [11]. Revision due to proximal catheter misplacement occurred in 1.6% of cases in the present cohort versus 4% in the freehand cohort [11].

The principal strength of this study lies in its design as the first prospective, randomized controlled trial directly comparing US-G and ST-G VPS placement. This pragmatic trial was embedded within standard departmental clinical practice, including routine preoperative and postoperative evaluations and follow-up at six weeks and six months.

### Limitations

This was a single-centre study, and although all surgical procedures followed standardized departmental protocols, variations in individual technique between surgeons cannot be fully excluded. The trial was conducted in a semi-blinded design: participants and neuroradiologists assessing secondary outcomes were blinded, whereas blinding of surgeons was not feasible. Because US-G VPS has been routinely implemented in our department only since 2019, its steep learning curve likely contributed to the higher number of ventricular puncture attempts observed in the US-G group.

The findings primarily reflect current surgical practice and patient characteristics within Switzerland and may be most applicable to similar European healthcare settings. Differences in institutional experience, equipment availability, or case mix could influence outcomes in other regions. Moreover, as most included participants had NPH, the results may not fully generalize to patients with alternative hydrocephalus aetiologies.

## Conclusions

Compared with ST-G, US-G VPS placement offers a faster, equally accurate, and safe alternative for ventricular catheter insertion. Its ease of use and procedural efficiency make it particularly suited for routine application in modern neurosurgical workflows. Broader adoption of US-G navigation could streamline VPS surgery and reduce overall procedural burden.

## Supplementary Information

Below is the link to the electronic supplementary material.

Supplementary Material 1: Additional File 1: Additional File 1.pdf, Trial Protocol, Statistical Analysis Plan

Supplementary Material 2: Additional File 2: Additional File 2.pdf, Composition of the primary outcome Surgical intervention time

Supplementary Material 3: Additional File 3: Additional File 3.pdf, Study visits and assessments

Supplementary Material 4: Additional File 4: Additional File 4.pdf, Sample size calculation

Supplementary Material 5: Additional File 5: Additional File 5.pdf, Surgical intervention time (min) and differences between groups

Supplementary Material 6: Additional File 6: Additional File 6.pdf, Operation time (Linear regression)

Supplementary Material 7: Additional File 7: Additional File 7.pdf, Operation time (min) and differences between groups

Supplementary Material 8: Additional File 8: Additional File 8.pdf, Anaesthesia time (Linear regression)

Supplementary Material 9: Additional File 9: Additional File 9.pdf, Anesthesia time (min) and differences between groups

Supplementary Material 10: Additional File 10: Additional File 10.pdf, Ventricular puncture attempts (Poisson regression), multiple attempts needed (Logistic regression)

Supplementary Material 11: Additional File 11: Additional File 11.pdf, Catheter position (Logistic regression)

Supplementary Material 12: Additional File 12: Additional File 12.pdf, Catheter position grading (Ordinal logistic regression)

Supplementary Material 13: Additional File 13: Additional File 13.pdf, Shunt dysfunction (Logistic regression)

Supplementary Material 14: Additional File 14: Additional File 14.pdf, Revision surgery (Logistic regression) and infection-related revisions

Supplementary Material 15: Additional File 15: Additional File 15.pdf, Complications (Logistic regression)

Supplementary Material 16: Additional File 16: Additional File 16.pdf, Modified Rankin Scale (Fishers exact test)

Supplementary Material 17: Additional File 17: Additional File 17.pdf, Ventricle volumes (Linear regression) and ventricle volumes reduction (Ordinal logistic regression)

Supplementary Material 18: Additional File 18: Additional File 18.pdf, Evans index (Linear regression) and Evans index improvement (Ordinal logistic regression)

## Data Availability

Metadata from this study are available on Zenodo (( http://www.zenodo.org ), 10.5281/zenodo.17378539). The raw data are available upon reasonable request and subject to approval by the Data access committee of the Medical Faculty of the University of Basel. Researchers wishing to access the raw data should contact the Data Access Committee at [med-dac@unibas.ch](mailto: med-dac@unibas.ch). Access will be granted to qualified researchers for non-commercial research purposes, in accordance with ethical and legal requirements. The statistical codes can be assessed at GitHub (https://github.com/SORC-Basel/NAVPS_RCT).

## Abbreviations

AC-PC: Anterior commissure-posterior commissure
AI: Artificial intelligence
BMI: Body mass index
eCRF: (Electronic) case report form
CSF: Cerebrospinal fluid
CT: Computed tomography
EVD: External ventricular drain
GCS: Glasgow coma scale
GOS: Glasgow outcome scale
ICU: Intensive care unit
MR: Magnetic resonance
mRS: Modified rankin scale
NAVPS: Navigated ventriculoperitoneal shunt
NPH: Normal pressure hydrocephalus
OR: Operating room
ST-G: Stereotactically guided
US: Ultrasound
US-G: Ultrasound-guided
VPS: Ventriculoperitoneal shunt
