# Supplementary material for: Ultrasound-guided versus stereotactically navigated ventriculoperitoneal shunt placement: a randomized clinical trial
Source: Fluids Barriers CNS. 2026 Jun 26;23:85. doi: 10.1186/s12987-026-00833-2 (PMC13309968; doi:10.1186/s12987-026-00833-2)
Supplement: Supplementary file 1 — Supplementary Material 1: Additional File 1: Additional File 1.pdf, Trial Protocol, Statistical Analysis Plan [file 12987_2026_833_MOESM1_ESM.pdf]

Additional File 3: Study visits and assessments

Study Periods	Screening Admission	Treatment, Intervention Period			Follow-up	
**Visit**	1	2	3	4	5	6
**Time (hour, day, week)**	1 day preop.	Operation day	2-5 days post-op. (48-120h)	At discharge (approx. 7 days postop.)	6-8 weeks post-op.	6 months postop.
**Patient Information and Informed Consent**	X					
**Randomization**	X					
**Demographics (age, sex)**	X					
**In- /Exclusion Criteria**	X					
**Neurologic examination**	X		X	X	X	X
**cCT scan**	(stereotactic navigation group, 1 to 5 days preoperatively)		X (2nd to 5th day or earlier in case of neurologic symptoms)			X
**Medical history**	X					
**Primary outcome (Surgical intervention time)**		X				
**Secondary Outcomes**		X	X	X	X	X
**VPS dysfunction**		X	X	X	X	X
**Operative Revision and reasons**		X	X	X	X	X
**Operation and anaesthesia time**		X				
**Number of puncture attempts**		X				
**Complications**		X	X	X	X	X
**Hospitalisation time (days)**				X		
**Intensive care unit (ICU) time (days)**				X		
**Discharge destination**				X		
**Adverse events**		X	X	X	X	X
**Death**		X	X	X	X	X
