# Supplementary figures and images for: Ultrasound-guided versus stereotactically navigated ventriculoperitoneal shunt placement: a randomized clinical trial
Source: Fluids Barriers CNS. 2026 Jun 26;23:85. doi: 10.1186/s12987-026-00833-2 (PMC13309968; doi:10.1186/s12987-026-00833-2)

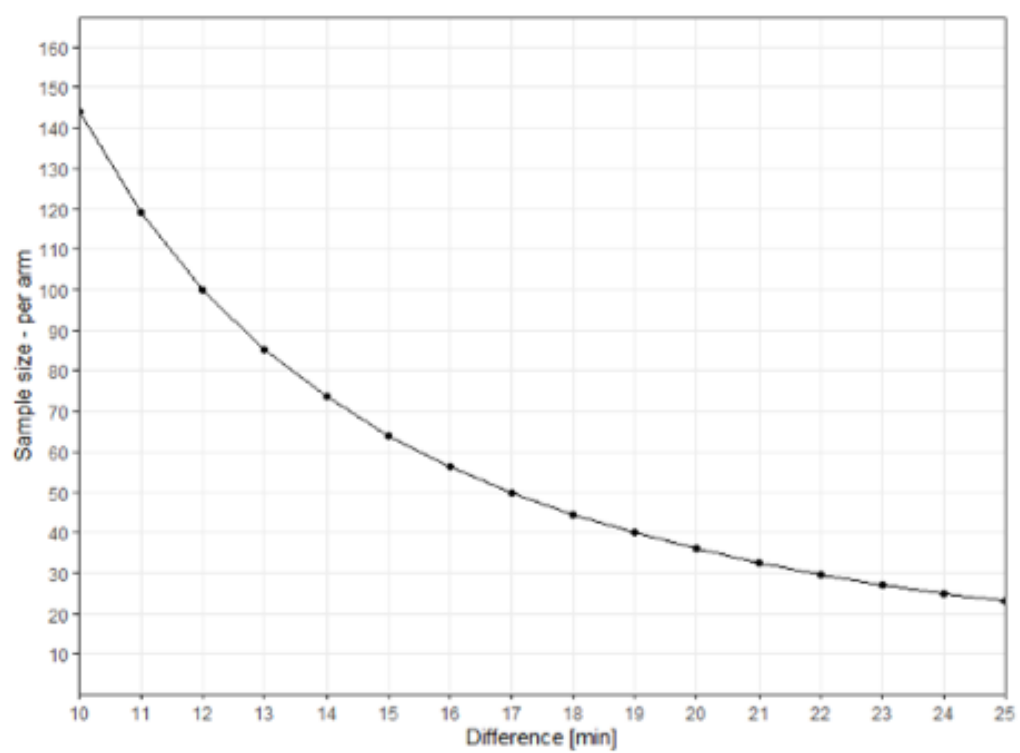

Supplement: Supplementary file 2 — Supplementary Material 2: Additional File 2: Additional File 2.pdf, Composition of the primary outcome Surgical intervention time [file 12987_2026_833_MOESM2_ESM.pdf]

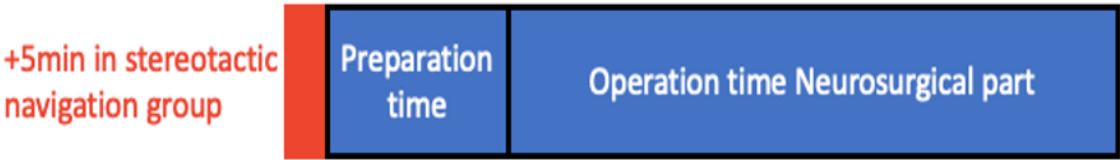

Supplement: Supplementary file 3 — Supplementary Material 3: Additional File 3: Additional File 3.pdf, Study visits and assessments [file 12987_2026_833_MOESM3_ESM.pdf]

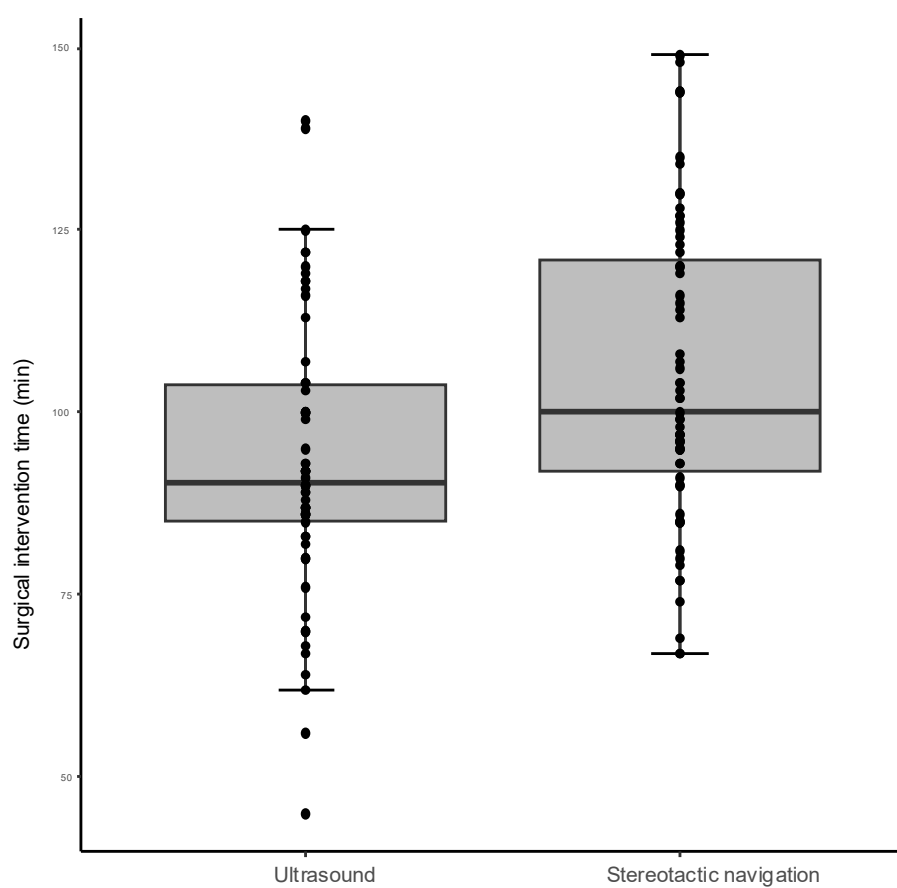

Supplement: Supplementary file 4 — Supplementary Material 4: Additional File 4: Additional File 4.pdf, Sample size calculation [file 12987_2026_833_MOESM4_ESM.pdf]

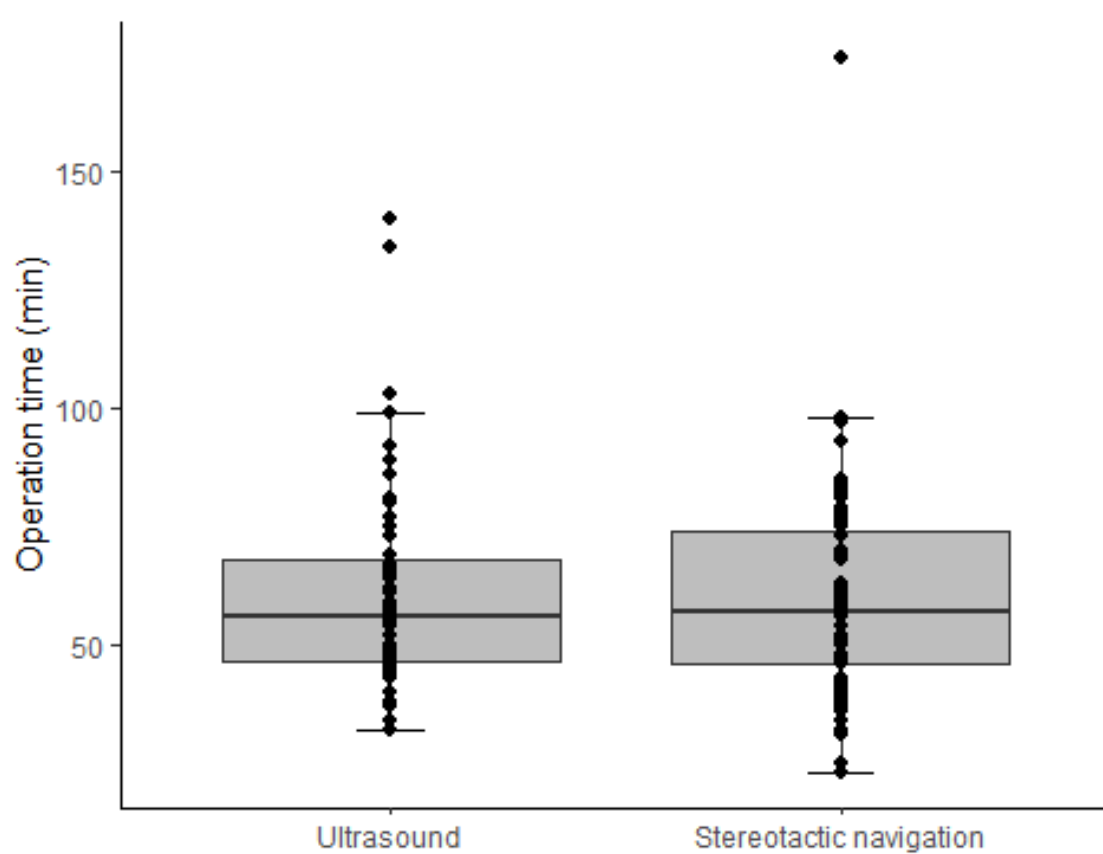

Supplement: Supplementary file 8 — Supplementary Material 8: Additional File 8: Additional File 8.pdf, Anaesthesia time (Linear regression) [file 12987_2026_833_MOESM8_ESM.pdf]

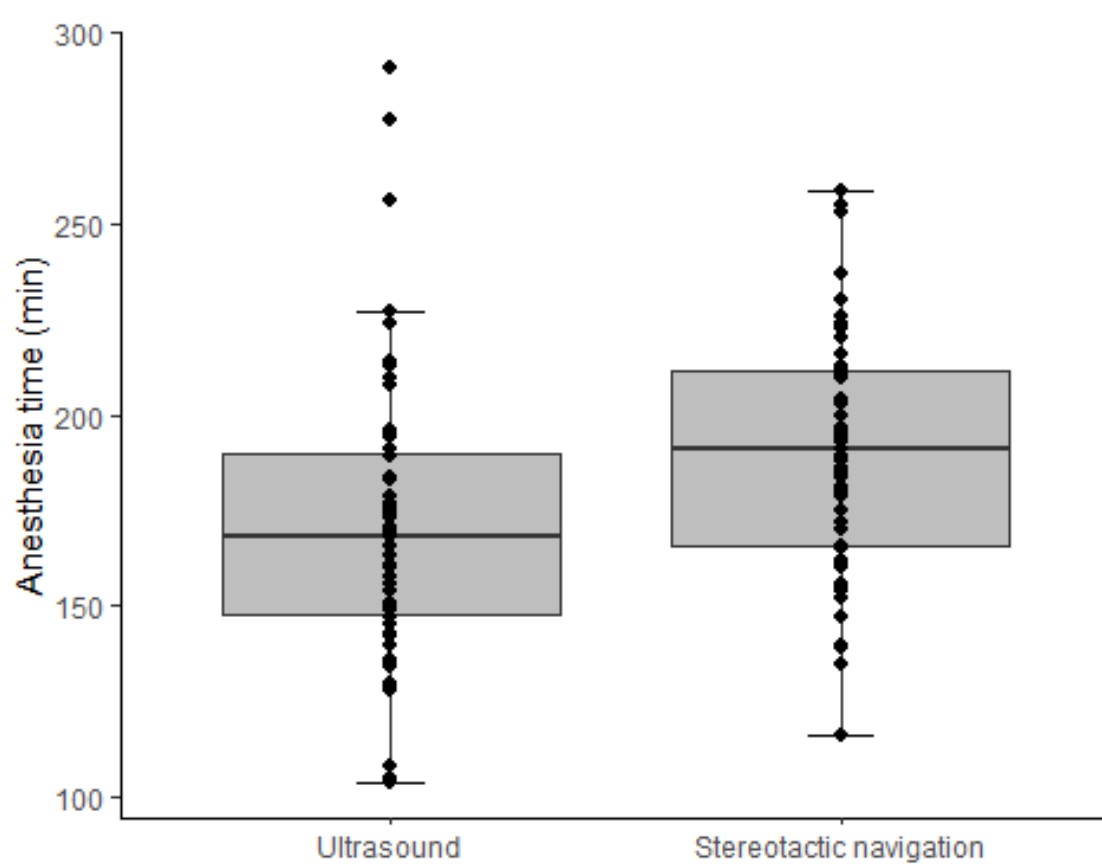

Supplement: Supplementary file 10 — Supplementary Material 10: Additional File 10: Additional File 10.pdf, Ventricular puncture attempts (Poisson regression), multiple attempts needed (Logistic regression) [file 12987_2026_833_MOESM10_ESM.pdf]
