# Supplementary material for: Ultrasound-guided versus stereotactically navigated ventriculoperitoneal shunt placement: a randomized clinical trial
Source: Fluids Barriers CNS. 2026 Jun 26;23:85. doi: 10.1186/s12987-026-00833-2 (PMC13309968; doi:10.1186/s12987-026-00833-2)
Supplement: Supplementary file 5 — Supplementary Material 5: Additional File 5: Additional File 5.pdf, Surgical intervention time (min) and differences between groups [file 12987_2026_833_MOESM5_ESM.pdf]

Additional File 1:

- Trial Protocol (Version 3.4, 20.08.2024)
- Statistical Analysis Plan (Version 1.4, 08.04.2025)

Intraoperative Ultrasound Guided compared to Stereotactic Navigated Ventriculoperitoneal Shunt Placement: A Randomized Controlled Study

Short title: Navigated VP-Shunt-Study (NaVPS-Study)

Clinical Study Protocol

Study Type:	- Clinical trial with Investigational Medical Device (MD)
Study Categorisation:	- Risk category A
Study Registration:	- Anticipated registry: clinicaltrials.gov and www.kofam.ch
Study Identifier:	- NaVPS-Study
Sponsor, Sponsor-Investigator or Principal Investigator:	- Prof. Dr. med. Luigi Mariani<br>Chair Department of Neurosurgery<br>University Hospital of Basel<br>Spitalstr.21, 4031 Basel<br>Switzerland<br>-
Investigational Product:	- Ultrasound navigation: BK Medical 5000 with burr hole probe (Type 9063 N11C5S, 11-5MHz)<br>Stereotactic navigation: Brainlab Dual Curve System (cranial navigation software version 3.1.)
Protocol Version:	- Version 3.4
Date:	- 20/08/2024<br>-

CONFIDENTIAL

The information contained in this document is confidential and the property of Prof. Luigi Mariani (or “sponsor”). The information may not - in full or in part - be transmitted, reproduced, published, or disclosed to others than the applicable Competent Ethics Committee(s) and Regulatory Authority(ies) without prior written authorisation from the sponsor except to the extent necessary to obtain informed consent from those who will participate in the study.

Ultrasound Guided compared to Stereotactic Navigated Ventriculoperitoneal Shunt Placement: A Randomized Controlled Study (NaVPS-Study)

Statistical analyses plan

Project number NCH-003

Analysis for Prof. Dr. med. Jehuda Soleman and Dr. med. Severina Leu

Department of Neurosurgery, University Hospital Basel

Authored by Florian Halbeisen, Surgical Outcome Research Center

Email: floriansamuel.halbeisen@usb.ch

Reviewed by Brigitta Gahl, Surgical Outcome Research Center

Email: brigitta.gahl@usb.ch

Version: 1.4 – 08.04.2025

SORC
SURGICAL OUTCOME RESEARCH CENTER

Inhalt

1. Introduction ... 3
   1.1. Background and rationale ... 3
   1.2. Objectives ... 3
2. Study methods ... 3
   2.1. Trial design ... 3
   2.2. Randomisation ... 3
   2.3. Sample size ... 3
   2.4. Framework ... 3
   2.5. Stratification ... 4
   2.6. Statistical interim analyses and stopping guidance ... 4
3. Data management ... 4
   3.1. Data export ... 4
   3.2. Data validation ... 4
4. Statistical principles ... 4
   4.1. General ... 4
   4.2. Confidence intervals and p-values ... 4
   4.3. Adherence and protocol deviations ... 4
   4.4. Analysis populations ... 4
5. Trial Population ... 5
   5.1. Screening data ... 5
   5.2. Eligibility ... 5
   5.3. Patient flow ... 5
   5.4. Withdrawal/follow-up ... 5
   5.5. Baseline patient characteristics ... 5
6. Analysis ... 5
   6.1. Outcome definitions ... 5
   6.2. Outcome derivation ... 6
   6.3. Analysis methods ... 6
      6.3.1 Primary analysis ... 6
   6.4. Missing data ... 7
   6.5. Evaluation of safety parameters ... 7
   6.6. Statistical software ... 7
7. Changes from the protocol ... 7
   7.1. Interims analysis ... 8
8. References ... 7

1. Introduction

1.1. Background and rationale

See study protocol.

1.2. Objectives

The objective of this study is to analyse the feasibility and safety of Ultrasound-guided (US-G) Ventriculoperitoneal shunt (VPS) placement. The primary objective of the study is to compare the surgical intervention of VPS placement between US-G and stereotactic navigated placement plus additional 5 minutes.

The Null-hypothesis is that there are no differences in the surgical intervention time (plus additional 5 minutes in the stereotactic navigation group) between the two methods. The Alternative Hypothesis is that US-G VPS placement has a shorter surgical intervention time compared to stereotactic navigated VPS placement.

$$H_0: Time_{\ US-G} = Time_{\ \mathrm{Stereotactic}} + 5\mathrm{min}$$

$$H_1: Time_{\ US-G} < Time_{\ \mathrm{Stereotactic}} + 5\mathrm{min}$$

2. Study methods

2.1. Trial design

This study is a prospective randomized two-arms controlled superiority trial, conducted in a primary neurosurgical centre in Switzerland.

2.2. Randomisation

Randomisation will be performed by an independent individual using a stratified simple randomisation procedure as implemented in the electronic data capture software REDCap. An allocation ratio of 1:1 will ensure a balance in sample size across both groups over time. The randomisation will be stratified according to age of the patients (under 40 years/over 40 years).

2.3. Sample size

The sample size was estimated with the aim of showing a surgical intervention time reduction of 15 minutes (min) in the intervention arm compared to the control arm. The significance level was chosen to be 5%, while the power was chosen to be (1-β) = 80%.

Based on unpublished data from a pilot study assessing the surgical intervention time in patients undergoing VPS placement using US navigation, we calculated a mean surgical intervention time of 63 min with a standard deviation (SD) of 28.65 min. Because of the additional steps involved using stereotactically navigated surgery, we assume an increased surgical invention time in those patients.

We then conservatively selected a scenario with a relatively modest difference between groups. This resulted in 58 patients with VPS placement surgery per group. Anticipating a 1% drop-out by deaths and a surgeons' non-adherence rate (change from control to treatment arm) of 5%, a total sample size of 130 patients (65 per group) was required.

2.4. Framework

All endpoints will be analysed for the superiority of US-G VPS placement compared to stereotactic navigated VPS placement.

2.5. Stratification

Unless explicitly mentioned, analyses will not be stratified.

2.6. Statistical interim analyses and stopping guidance

No interim analysis planned.

3. Data management

3.1. Data export

The entered data will be exported from the trial database (REDCap) to a statistical software package.

3.2. Data validation

Data validation and cleaning will be conducted after completion of data entry but before database lock.

4. Statistical principles

4.1. General

All recorded and derived variables will be presented by intervention group (and visits, if appropriate) using descriptive summary tables. Continuous variables will be summarised by mean and standard deviation, or median and quartiles. Categorical variables will be summarised with absolute and relative frequencies.

4.2. Confidence intervals and p-values

The statistical testing will be two-sided with a significance level of 5%. All tests will be accompanied by an effect measure with a 95% confidence interval (95% CI).

4.3. Adherence and protocol deviations

Substantial amendments are changes that affect the safety, health, rights and obligations of participants, changes in the protocol that affect study objective(s) or central research topic, changes of study site(s) or of study leader and sponsor (ClinO, Art. 29).

4.4. Analysis populations

The population for analysis will comprise all patients as randomized and with completed follow-up (no drop-outs), hence the full analysis set (FAS). All statistical analyses will be performed on the FAS according to the intention-to-treat principle (i.e. all participants will be analysed on the basis of the intervention to which they were randomly allocated).

The per-protocol (PP) set will include all participants in the FAS who fulfilled the eligibility criteria, for whom the surgery was completed as planned in the study protocol, and for whom the measurement of the primary outcome is available.

5. Trial Population

5.1. Screening data

Screening data is not collected in the eCRF but the number of screened patients will be calculated from screening logs.

5.2. Eligibility

Inclusion and exclusion criteria are defined in the study protocol. Data about reason for non-inclusion is not documented in the eCRF and will not be available.

5.3. Patient flow

A CONSORT patient flow diagram will be drawn following the CONSORT 2010 standards.[1]

5.4. Withdrawal/loss to follow-up/drop-outs

All withdrawals, drop-outs and losses to follow-up will be listed with time points and reasons (if available).

5.5. Baseline patient characteristics

Evaluations of the baseline characteristics will be based on the FAS data set. They will be presented in a descriptive summary table by intervention group. Continuous variables will be shown as mean and standard deviation or median and quartiles, categorical variables as absolute and relative frequencies. No statistical comparisons of patient characteristics at baseline will be performed.

6. Analysis

Baseline characteristics:
Preoperative patient data such as age, sex, BMI (body mass index), neurologic examination (GCS, modified Rankin Scale (mRS), Glasgow Outcome Scale (GOS), neurological symptoms (headaches, vomitus, coma, gait disturbances, dementia, urinary incontinence, motor deficit, sensory deficit, aphasia, delir, others), underlying disease causing hydrocephalus, will be collected and summarised for both groups.

6.1. Outcomes

Primary outcomes:
- Surgical intervention time («Beginn oper. Vorbereitung (Lagerung)» to «Naht neurochirurgischer Teil») in minutes, plus additional 5 minutes in the stereotactic navigation group

Secondary outcomes:
- Operation time in minutes (time in minutes from “Schnitt” to “Naht”)
- Anaesthesia time in minutes (time in minutes from “Beginn Anästhesie” to “Ende Anästhesie”)
- Number of puncture attempts
- Catheter placement (optimal vs. not optimal, grade I to IV)
- Volumetry of side ventricles pre- and postoperatively in cm^3 (number and relative change)
- Evan’s Index[2] pre- and postoperatively (number and relative change)
- Complications (infection, bleeding, complications ass. with navigation method), mortality
- Revision surgery (yes/no) and reason for revision

Outcome definitions :
The primary endpoint is the surgical intervention time. This is the time spent in the OR by the surgeon and includes the preparation time together (patient positioning, head clamping in the steretocatic navigation group) with the operation time of the neurosurgical part. Beginning ("Lagerung") and ending ("Naht neurosurgical part") of this time interval is clearly defined and will be in a standardized manner recorded by blinded anaesthesiologists. In the stereotactic navigation group, an additional 5 minutes will be added to the surgical intervention time for the preplanning of the navigation the day before the operation on the Brainlab workstation.

The optimal catheter placement has been defined as free-floating within the CSF without touching the ventricle wall or septum pellucidum, and the tip of the catheter located at the foramen of Monro showing an optimal length of the catheter. Catheters that did not fulfill all criteria have been defined as not optimally placed. Positioning of catheters will be further graded according to Yim et al. into grades I to IV (grade I: catheter terminates in the ipsilateral frontal horn, grade II: catheter terminates in contralateral frontal horn, grade III: catheter terminates in non-targeted CSF spaces, grade IV: catheter terminates intraparenchymally)[3].

6.3. Analysis methods

6.3.1 Primary analysis

Analysis of the primary endpoint will follow the intention-to-treat (ITT) principle. It will be based on the FAS with missing data of the primary outcome imputed as described in the section "Missing data" (section 6.4). Patient data will be analysed according to their intervention allocation, irrespective of actual treatment received.

The primary outcome, surgical intervention time, will be assessed using a linear regression model. The estimates will be reported with 95% confidence intervals (CI). Explanatory variables will be BMI, underlying diagnosis, experience surgeon and the use of an old burr hole.

In case the outcome variable is not normally distributed, data will be transformed or a different model, depending on the distribution of the data, chosen.

6.3.1.2. Analysis of secondary outcomes

All analysis of the secondary endpoints will follow will follow the intention-to-treat (ITT) principle. It will be based on the FAS with missing data of the primary outcome imputed as described in the section "Missing data" (section 6.4).

The continuous secondary endpoints (outcomes operation time, anaesthesia time, volumetry of side ventricles, and Evan's Index) will be assessed using linear regression models, the binary endpoints (optimal catheter placement, complications, mortality and revision surgery) will be assessed using logistic regression, and the number of puncture attempts will be assessed using a poisson regression model.

All estimates will be reported with 95% CI. IF numbers allow explanatory variables will be be BMI, underlying diagnosis, experience surgeon and the use of an old burr hole.

In case the outcome variable is not normally distributed or model assumptions are violated, data will be transformed or a different model, depending on the distribution of the data, chosen.

Deviation from the original statistical plan:
If substantial deviations of the analysis as outlined in these sections are needed for whatever reason, the protocol will be amended. All deviations of the analysis from the protocol or from the detailed analysis plan will be listed and justified in a separate section of the final statistical report.

6.4. Missing data

Missing baseline and outcome data will be summarized by study arm. As outlined above, the primary analyses will be the intent-to-treat population. In the case of missing data or drop-outs, we may adjust for further baseline variables which are associated with missing outcome data[5] or we consider multiple imputation. Both, baseline patient characteristics and outcome variables will be considered for multiple imputations. Variables with more than 50% missing values will not be used for the imputation model. Categorical variables with a frequency of 5% or less in one category will also be omitted. Continuous variables will be imputed using linear regression and binary variables using logistic regression. We will construct and analyze 20 imputed data sets and combine results using Rubin's rules.[2] Diagnostic checks, including model convergence and comparing observed versus imputed distributions, will be performed to ensure the quality of imputations.

6.5. Evaluation of safety parameters

Evaluation of safety parameters will be based on the safety population (FAS, section 4.4). They will be listed according to the treatment the patient actually received with the time points of onset. If many adverse events should be observed, we will compare frequency between treatment groups.

Safety endpoints are the following events: positioning of the catheter, number of ventricle puncture attempts, VPS dysfunction, revision rate, perioperative complications (bleeding, infection), complications due to head clamp, death, and coma.

6.6. Statistical software

The statistical analysis will be performed by Surgical Outcome Research Center using the statistical software R (Version 4.3.2, The R Foundation for Statistical Computing, Vienna, Austria).

7. Changes from the protocol

None.

8. References

1. http://www.consort-statement.org/consort-2010

2. Evans WJ. An encephalographic ratio for estimating ventricular enlargement and cerebral atrophy. Archives of Neurology & Psychiatry. 1942;47:931-937.

3. Yim B, Reid Gooch M, Dalfino JC, Adamo MA, Kenning TJ. Optimizing ventriculoperitoneal shunt placement in the treatment of idiopathic intracranial hypertension: an analysis of neuroendoscopy, frameless stereotaxy, and intraoperative CT. Neurosurg Focus. 2016;40(3):E12

4. Sullivan TR, White IR, Salter AB, Ryan P, Lee KJ. Should multiple imputation be the method of choice for handling missing data in randomized trials? Stat Methods Med Res. 2018;27(9):2610-2626.

5. Rubin DB. Multiple imputation for nonresponse in surveys. New York: John Wiley & Sons; 2004.

Signature Pages

Study number	-	2019-02157
Study Title	-	Prospective randomized comparison of stereotactic navigated versus ultrasound navigated ventriculoperitoneal shunt placement
	-	

The Sponsor-Investigator and trial statistician have approved the amended protocol version [3.0 (06/07/2022)], and confirm hereby to conduct the study according to the protocol, current version of the World Medical Association Declaration of Helsinki[1], ICH-GCP[2,3] guidelines or ISO 14155[4] norm if applicable and the local legally applicable requirements.

Sponsor-Investigator:

Prof. Dr. med. Luigi Mariani
Chair Department of Neurosurgery, University Hospital of Basel, Spitalstr. 21, 4031 Basel,

____________________ ____________________

Place/Date Signature

Local Principal Investigator at study site:

I have read and understood this trial protocol and agree to conduct the trial as set out in this study protocol, the current version of the World Medical Association Declaration of Helsinki, ICH-GCP[2,3] guidelines or ISO 14155[4] norm and the local legally applicable requirements.

Site	Department of Neurosurgery, University Hospital of Basel, Spitalstr. 21, 4031 Basel, Switzerland<br>-
Principal investigator	- Dr. med. Severina Leu

Place/Date Signature

Co-Investigator - PD Dr. med. Jehuda Soleman

Place/Date Signature

Trial Statistician:

Florian Halbeisen, PhD
Basel Institute for Clinical Epidemiology & Biostatistics, University Hospital Basel, Spitalstrasse 12, 4031 Basel, Switzerland

Place/Date Signature

Table of Contents

STUDY SYNOPSIS **7**
ABBREVIATIONS **11**
STUDY SCHEDULE **12**
Discharge destination 12
1. STUDY ADMINISTRATIVE STRUCTURE **13**
1.1 Sponsor, Sponsor-Investigator 13
1.2 Principal Investigator 13
1.4 Statistician ("Biostatistician") 13
1.5 Monitoring institution 13
1.6 Any other relevant Committee, Person, Organisation, Institution 13
2. ETHICAL AND REGULATORY ASPECTS **13**
2.1 Study registration 14
2.2 Categorisation of study 14
2.3 Competent Ethics Committee (CEC) 14
2.4 Ethical Conduct of the Study 14
2.5 Declaration of interest 14
2.6 Patient Information and Informed Consent 14
2.7 Participant privacy and confidentiality 14
2.8 Early termination of the study 15
2.9 Protocol amendments 15
3. BACKGROUND AND RATIONALE **16**
3.1 Background and Rationale 16
3.2 Investigational Product (treatment, device) and Indication 16
3.3 Preclinical Evidence 16
3.4 Clinical Evidence to Date 16
3.5 Dose Rationale / Medical Device: Rationale for the intended purpose in the study (pre-market MD) 16
3.6 Explanation for the choice of the comparator (or placebo) 17
3.7 Benefits / Risks 17
3.8 Justification of choice of the study population 17
4. STUDY OBJECTIVES **18**
4.1 Overall Objective 18
4.2 Primary Objective 18
4.3 Secondary Objectives 18
4.4 Safety Objectives 18
5. STUDY OUTCOMES **19**
5.1 Primary Outcome 19
5.2 Secondary Outcomes 19
5.3 Other Outcomes of Interest 19
5.4 Safety Outcomes 19
6. STUDY DESIGN **20**
6.1 General study design and justification of design 20
6.2 Methods of minimising bias 21
6.2.1 Randomisation 21
6.2.2 Blinding procedures 21

6.2.3 Other methods of minimising bias 21
6.3 Unblinding Procedures (Code break) 21
7 STUDY POPULATION 22
7.1 Eligibility criteria 22
7.2 Recruitment and screening 22
7.3 Assignment to study groups 22
7.4 Criteria for withdrawal/discontinuation of participants 22
8 STUDY INTERVENTION 23
8.1 Identity of Investigational Products (treatment / medical device) 23
8.1.1 Experimental Intervention (treatment / medical device) 23
8.1.2 Control Intervention (standard/routine/comparator treatment / medical device) 23
8.1.3 Packaging, Labelling, and Supply (re-supply) 23
8.1.4 Storage Conditions 23
8.2 Administration of experimental and control interventions 23
8.2.1 Experimental Intervention 23
24
8.2.2 Control Intervention 24
8.3 Dose / Device modifications 24
8.4 Compliance with study intervention 24
8.5 Data Collection and Follow-up for withdrawn participants 25
8.6 Trial specific preventive measures 25
8.7 Concomitant Interventions (treatments) 25
8.8 Study Drug / Medical Device Accountability 25
8.9 Return or Destruction of Study Drug / Medical Device 25
9 STUDY ASSESSMENTS 26
9.1 Study flow chart / table of study procedures and assessments 26
Discharge destination 26
9.2. Assessments of outcomes 27
9.2.1. Assessment of primary outcome 27
27
Figure 1: Composition of the surgical intervention time (preparation time plus operation time neurosurgical part), plus additional five minutes in the stereotactic navigation group. 27
9.2.2. Assessment of secondary outcomes 27
9.2.3 Assessment of other outcomes of interest 27
9.2.4 Assessment of safety outcomes 27
9.2.5 Assessments in participants who prematurely stop the study 28
9.3 Procedures at each visit 28
9.3.1 Visit 1: Screening and admission 28
9.3.2 Visit 2: Operation day 28
9.3.3 Visit 3: 2nd to 5th postoperative day (48-120h postoperative) 28
9.3.4 Visit 4: Discharge (approx. 7 days postoperatively) 28
9.3.5 Visit 5: 1st Follow-up (6-8 weeks postoperatively) 28
9.3.5 Visit 6: 2nd Follow-up (6 months postoperatively) 29
10 SAFETY 30
10.3 Medical Device Category A studies 30
10.3.1 Definition and Assessment of safety related events 30

10.3.2 Reporting of Safety related events 31
11 STATISTICAL METHODS 33
11.1 Hypothesis 33
11.2 Determination of Sample Size 33
11.3 Statistical criteria of termination of the trial 33
11.4 Planned Analyses 33
11.4.1 Datasets to be analysed, analysis populations 34
11.4.2 Primary Analysis 34
11.4.3 Secondary Analyses 34
11.4.4 Interim analyses 34
11.4.5 Safety analysis 34
11.4.6 Deviation(s) from the original statistical plan 34
11.5 Handling of missing data and drop-outs 34
12 QUALITY ASSURANCE AND CONTROL 35
12.1 Data handling and record keeping / archiving 35
12.1.1 Case Report Forms 35
12.1.2 Specification of source documents 35
12.1.3 Record keeping / archiving 35
12.2 Data management 35
12.2.1 Data Management System 35
12.2.2 Data security, access and back-up 35
12.2.3 Analysis and archiving 36
12.2.4 Electronic and central data validation 36
12.3 Monitoring 36
12.4 Audits and Inspections 36
12.5 Confidentiality, Data Protection 36
12.6 Storage of biological material and related health data 36
13 PUBLICATION AND DISSEMINATION POLICY 37
14 FUNDING AND SUPPORT 38
14.1 Funding 38
14.2 Other Support 38
N/A 38
15 INSURANCE 39
APPENDICES 40
REFERENCES 41

STUDY SYNOPSIS

**Sponsor / Sponsor-Investigator**	- Prof. Dr. med. Luigi Mariani,<br>- Chair Department of Neurosurgery, University Hospital of Basel<br>- Spitalstr. 21, 4031 Basel<br>-
**Study Title:**	- Intraoperative Ultrasound Guided compared to Stereotactic Navigated Ventriculoperitoneal Shunt Placement: A Randomized Controlled Study
**Short Title / Study ID:**	- Navigated VP-Shunt-Study (NaVPS-Study)<br>- BASEC No 2019-02157
**Protocol Version and Date:**	- Version 3.4<br>- 20.08.2024
**Trial registration:**	- ClinicalTrials.gov: NCT04450797<br>- Swiss Federal Complementary Database (https://www.kofam.ch/de/studienportal/suche/88135/studie/49552)
**Study category and Rationale**	- Risk category A
**Background and Rationale:**	- Ventriculoperitoneal shunt (VPS) placement is one of the most frequent procedures in neurosurgical practice and is done approximatively once per week in our clinic (40 to 50 cases per year). The indications vary from normal pressure hydrocephalus in elderly patients to VPS-dependency after subarachnoid haemorrhage, infection or trauma, mostly in younger patients[5-7]. The position of the proximal ventricular catheter is important since it influences possible malfunction of the VPS[8-11]. With freehand placement rates of malpositioned VPS are still high[12].<br><br>For the improvement of accuracy in proximal VPS placement, navigation-based insertion techniques have been developed. VPS placement using stereotactic navigation has shown a high accuracy of catheter placement, while the main limitations are that for referencing, the head of the patient needs to be fixed in a head holder and the preoperative set-up can be time-consuming[13]. Ultrasound-guided (US-G) VPS placement using a burr hole probe was described as an alternate for image-guided VPS placement technique[14]. For US-G VPS placement head fixation or preoperative registration is not needed. Initially, the entry point is measured according to standard anatomical landmarks, thereafter the catheter is placed into the ventricle under real-time US guidance along the depicted trajectory[14]. To date, most of the clinical evidence regarding image-guided VPS placement derives from small retrospective cohort studies [15-17].<br><br>Installation and use of image guidance techniques add extra time to the standardized procedure of VPS placement, leading to longer operation times, and higher costs. Beside that patients will be anasesthezized for longer time, leading to possible complications of prolonged anesthesia. An ideal image guidance technique should therefore be easy to handle, and not too time consuming.<br><br>- The rationale of this study is to prospectively compare US-G VPS placement to stereotactic navigation in a randomized controlled fashion with the surgical intervention time as primary outcome.
**Objective(s):**	The main objective of this study is to analyse the feasibility and safety of US-G VPS placement. Furthermore, we are interested in different safety aspects of both methods. This study is designed as a prospective randomized trial comparing the outcomes of US-G VPS placement to stereotactic navigated placement.

**Outcome(s):**	**Primary study outcome measures**:<br>- Surgery intervention time («Beginn oper. Vorbereitung (Lagerung)» to «Naht neurochirurgischer Teil») in minutes, plus additional 5 minutes in the stereotactic navigation group<br>**Secondary study outcome measures**:<br>- Operation time in minutes<br>- Anaesthesia time in minutes<br>- Number of puncture attempts<br>- Catheter placement (optimal vs. not optimal, grade I to IV)<br>- Volumetry of side ventricles pre- and postoperatively in cm^3 (number and relative change)<br>- Evan's Index pre- and postoperatively (number and relative change)<br>- Complications (infection, bleeding, complications ass. with navigation method), mortality<br>- Revision surgery (yes/no) and reason for revision
**Study design:**	- Prospective, randomized, controlled study for analysis of feasibility and safety<br>- 1:1 randomization
**Inclusion criteria:**<br><br><br><br><br>**Exclusion criteria:**	- Patients undergoing VPS placement<br>- Patients at the age of 18 years or older<br>- Informed Consent as documented by signature<br>- (Appendix Informed Consent Form)<br><br>- Patients under the age of 18 years<br>- No informed consent.<br>- Emergency surgery if there is no time for installation of any navigation system<br>- Revision surgery using the same side and location, or revision surgery where no completely new shunt will be placed<br>- Ventriculoatrial and ventriculopleural placement<br>- Women who are pregnant or breast feeding<br>- Intention to become pregnant during the course of the study<br>- Enrolment of the investigator, his/her family members, employees, and other dependent persons<br>-
**Randomisation:**	- 1:1 randomisation, stratified according to age of the patients (under 40 years/over 40 years)<br>- Randomisation via REDCap usually one day preoperatively (elective surgery), or directly preoperative (semi-emergency surgery)

**Measurements and procedures:**	- Admission:<br>> Randomisation, demographics (age, gender), height, weight, body mass index (BMI)<br>> neurologic examination (GCS, modified Rankin Scale (mRS), Glasgow Outcome Scale (GOS), neurological symptoms (headaches, vomitus, coma, gait disturbances, dementia, urinary incontinence, motor deficit, sensory deficit, aphasia, delir, others)<br>> medical history (underlying disease causing hydrocephalus (NPH, SAH, IVH, other type of bleeding, trauma, tumor, congenital, other), prior EVD, prior VPS, or prior head operations including details)<br>> cCT scan (US group not older than 90 days, stereotactic navigation group needs a new CT scan at admission, measurements: Evans' index[18], volumetry of side ventricles)<br><br>Operation (day 0):<br>> Primary outcome (surgical intervention time)<br>> shunt side (right, left), shunt location (frontal, occipital), ventricular catheter length, number of surgeons, experience of main surgeon, shunt/valve manufacturer, type of valve (adjustable, non-adjustable), valve pressure, number of accessory incisions,<br>> shunt dysfunction, revisions surgery, indication for revision,<br>> secondary outcomes (operation time, anaesthesia time, number of puncture attempts, complications including complication details)<br>> death including reason of death<br><br>Postoperative during hospital stay (48-120hours):<br>> Neurologic examination (GCS, modified Rankin Scale (mRS), Glasgow Outcome Scale (GOS), neurological symptoms (headaches, vomitus, coma, gait disturbances, dementia, urinary incontinence, motor deficit, sensory deficit, aphasia, delir), neurology better, headaches better, vomitus better, gait ataxia better, dementia better, urinary incontinence better)<br>> cCT scan (catheter position (optimal vs. not optimal, grade I to IV), Evans' index, volumetry of side ventricles, Evans' Index improvement, ventricle width reduction)<br>> shunt dysfunction including details (proximal/distal obstruction, proximal/distal dislocation, abdominal cause, dysfunction due to infection, disconnection, other)<br>> revision surgery and indication for revision (bleeding, infection, obstruction, misplacement, disconnection, proximal/distal dislocation, other)<br>> complications other than dysfunction (infection, blee,ding, seizure, fracture, other)<br>> death including reason of death<br><br>Discharge:<br>> Neurologic examination (GCS, modified Rankin Scale (mRS), Glasgow Outcome Scale (GOS), neurological symptoms (headaches, vomitus, coma, gait disturbances, dementia, urinary incontinence, motor deficit, sensory deficit, aphasia, delir), neurology better, headaches better, vomitus better, gait ataxia better, dementia better, urinary incontinence better)<br>> shunt dysfunction including details (proximal/distal obstruction, proximal/distal dislocation, abdominal cause, dysfunction due to infection, disconnection, other)<br>> revision surgery and indication for revision (bleeding, infection, obstruction, misplacement, disconnection, proximal/distal dislocation, other)<br>> complications other than dysfunction (infection, blee,ding, seizure, fracture, other)<br>> duration of (postoperative) hospitalisation in days, duration of ICU stay in days<br>> discharge destination (home, rehabilitation, nursing facility, other hospital, other)<br>> death including reason of death<br><br>1. Follow-up (6-8 weeks postoperatively):<br>> Time of follow-up (date and number of days postoperative)<br>> neurologic examination (GCS, modified Rankin Scale (mRS), Glasgow Outcome Scale (GOS), neurological symptoms (headaches, vomitus, coma, gait disturbances, dementia, urinary incontinence, motor deficit, sensory deficit, aphasia, delir), neurology better, headaches better, vomitus better, gait ataxia better, dementia better, urinary incontinence better)<br>> shunt dysfunction including details (proximal/distal obstruction, proximal/distal dislocation, abdominal cause, dysfunction due to infection, disconnection, other)<br>> revision surgery and indication for revision (bleeding, infection, obstruction, misplacement, disconnection, proximal/distal dislocation, other)

	> complications other than dysfunction (infection, blee,ding, seizure, fracture, other)<br>> death including reason of death<br><br>2. Follow-up (6 months postoperatively):<br>> Time of follow-up (date and number of days postoperative)<br>> neurologic examination (GCS, modified Rankin Scale (mRS), Glasgow Outcome Scale (GOS), neurological symptoms (headaches, vomitus, coma, gait disturbances, dementia, urinary incontinence, motor deficit, sensory deficit, aphasia, delir), neurology better, headaches better, vomitus better, gait ataxia better, dementia better, urinary incontinence better)<br>> cCT scan (catheter position (optimal vs. not optimal, grade I to IV), Evans' index, volumetry of side ventricles, Evans' Index improvement, ventricle width reduction)<br>> shunt dysfunction including details (proximal/distal obstruction, proximal/distal dislocation, abdominal cause, dysfunction due to infection, disconnection, other)<br>> revision surgery and indication for revision (bleeding, infection, obstruction, misplacement, disconnection, proximal/distal dislocation, other)<br>> complications other than dysfunction (infection, bleeding, seizure, fracture, other)<br>- > death including reason of death
**Study Product / Intervention:**	- US -G VPS placement, done by BK Medical 5000 US with burr hole probe (type 9063 N11C5S, 11-5 MHz).<br>Head not fixed, placed on horseshoe head holder, no preoperative navigation planning, catheter will be cut in length after positioning under real-time US guidance.
**Control Intervention:**	- Stereotactic navigation for VPS placement (Brainlab Dual Curve System with cranial navigation software version 3.1).<br>- Head fixed in head clamp, entry point, trajectory and catheter length planned based on preoperative computer tomography imaging, catheter placed using navigated stylet.
**Number of Participants with Rationale:**	- Total: 130 + 6 = 136<br>Each group: 68<br>The sample size was estimated with the aim of showing a surgical intervention time reduction of 15 min. Based on data from a pilot study we assumed a standard deviation (SD) in surgical intervention time of 28.65 min. The significance level was chosen to be 5%, while the power was chosen to be (1-β) = 80%.<br>- For the final estimation we anticipating a drop-out rate of 1% and a surgeons' non-adherence rate (change of treatment arm) of 5%.
**Study Duration:**	Estimated duration 5 years (61 months)
**Study Schedule:**	- 01/2020 of First-Participant-In (planned)<br>- 02/2025 of Last-Participant-Out (planned)
**Investigators:**	- Dr. med. Severina Leu<br>Dr. med. Jehuda Soleman<br>- Klinik für Neurochirurgie, Spitalstr. 21, 4031 Basel
**Study Centre(s):**	Single centre: Klinik für Neurochirurgie, Spitalstr. 21, 4031 Basel
**Statistical Considerations:**	- The primary outcome, surgical intervention time, will be assessed using a linear regression model, reporting adjusted mean differences between arms. The estimates will be reported with 95% confidence intervals (CI). The model will be adjusted for the most important risk factors.
**GCP Statement:**	- This study will be conducted in compliance with the protocol, the current version of the Declaration of Helsinki, the ICH-GCP[2,3] or ISO EN 14155[4] (as far as applicable) as well as all national legal and regulatory requirements.

ABBREVIATIONS

- Provide a list of abbreviations used on the protocol - to be completed

AE	Adverse Event
BASEC	Business Administration System for Ethical Committees, (https://submissions.swissethics.ch/en/)
BMI	Body mass Index
CA	Competent Authority (e.g. Swissmedic)
CEB	Basel Institute of Clinical Epidemiology
CEC	Competent Ethics Committee
CT	Computer tomography
CRF	Case Report Form
CSF	Cerebrospinal fluid
ClinO	Ordinance on Clinical Trials in Human Research *(in German: KlinV, in French: OClin, in Italian: OSRUm)*[19]
eCRF	Electronic Case Report Form
CTCAE	Common terminology criteria for adverse events
DSUR	Development safety update report
EDC	Electronic Data Capture
ER	Emergency Room
EVD	External Ventricular Drain
GCP	Good Clinical Practice
GCS	Glascow Coma Scale
GOS	Glasgow Outcome Scale
IB	Investigator’s Brochure
ICU	Intensive Car Unit
Ho	Null hypothesis
H1	Alternative hypothesis
HRA	Federal Act on Research involving Human Beings *(in German: HFG, in French: LRH, in Italian: LRUm)*[20]
IMP	Investigational Medicinal Product
IIT	Investigator-initiated Trial
ISO	International Organisation for Standardisation
ITT	Intention to treat
MD	Medical Device
MedDO	Medical Device Ordinance *(in German: MepV, in French: ODim)*[21]
mRS	Modified Rankin scale
OR	Operating Room
PI	Principal Investigator
SDV	Source Data Verification
SOP	Standard Operating Procedure
SPC	Summary of product characteristics
SUSAR	Suspected Unexpected Serious Adverse Reaction
TMF	Trial Master File
US	Ultrasound
US-G	Ultrasound guided
VP	Ventriculoperitoneal
VPS	Ventriculoperitoneal Shunt

STUDY SCHEDULE

Study Periods	Screening Admission	Treatment, Intervention Period			Follow-up	
Visit	1	2	3	4	5	6
Time (hour, day, week)	1 day preop.	Opera-tion day	2-5 days post-op. (48-120h)	At discharge (approx. 7 days postop.)	6-8 weeks post-op.	6 months postop. (150-210 days postop.)
Patient Information and Informed Consent	x					
Randomization	x					
Demographics (age, sex)	x					
In- /Exclusion Criteria	x					
Neurologic examination	x		x	x	x	x
cCT scan	(x stereotactic navigation group, 1 to 5 days preoperative)		x (2nd to 5th day or earlier if neurologic symptoms)			x
Medical history	x					
Primary outcome (Surgical intervention time)		x				
Secondary Outcomes		x	x	x	x	x
VPS dysfunction		(x)	x	x	x	x
Operative Revision and reasons		(x)	x	x	x	x
Operation and anaesthesia time		x				
Number of puncture attempts		x				
Complications		x	x	x	x	x
Hospitalisation time (days)				x		
ICU time (days)				x		
**Discharge destination**				**x**		
Adverse events		x	x	x	x	x
**Death**		**x**	**x**	**x**	**x**	**x**

1. STUDY ADMINISTRATIVE STRUCTURE

1.1 Sponsor, Sponsor-Investigator

Prof. Dr. med. Luigi Mariani
Chair Department of Neurosurgery
University Hospital of Basel
Spitalstrasse 21
CH-4031 Basel
Luigi.Mariani@usb.ch
Tel: 0041 61 328 71 24

1.2 Principal Investigator

Dr. med. Severina Leu
Department of Neurosurgery
University Hospital of Basel
Spitalstrasse 21
CH-4031 Basel
Severina.Leu@usb.ch
Tel: 0041 76 537 84 28

1.3. Co-Investigator

PD Dr. med. Jehuda Soleman
Department of Neurosurgery
University Hospital of Basel
Spitalstrasse 21
CH-4031 Basel
Jehuda.Soleman@usb.ch
Tel: 0041 78 623 44 88

1.4 Statistician ("Biostatistician")

Florian Halbeisen, PhD
Basel Institute of Clinical Epidemiology
University Hospital of Basel
Spitalstrasse 12
CH-4031 Basel
Floriansamuel.halbeisen@usb.ch
Tel: 0041 61 328 54 10

1.5 Monitoring institution

Julia Manzetti, Klaus Ehrlich
Clinical Monitor
Departement Klinische Forschung
University Hospital of Basel
Schanzenstrasse 55
CH-4031 Basel
julia.manzetti@usb.ch
Tel. 0041 61 556 56 26

1.6 Any other relevant Committee, Person, Organisation, Institution

Birsel Klein-Reesink
Study Coordinator
Department of Neurosurgery
University Hospital of Basel
Spitalstrasse 21
CH-4031 Basel
birsel.klein-reesink@usb.ch
Tel: 0041 61 328 79 23

2. ETHICAL AND REGULATORY ASPECTS

The decision of the CEC and foreign competent authority concerning the conduct of the study will be made in writing to the Sponsor-Investigator before commencement of this study. The clinical study can only begin once approval

from all required authorities has been received. Any additional requirements imposed by the authorities shall be implemented.

2.1 Study registration

The study will be registered at clinicaltrials.gov and in the Swiss Federal Complementary Database.

2.2 Categorisation of study

This is a Category A study as the medical devices used in the study both bear conformity marking and they are used in accordance with the instructions.

2.3 Competent Ethics Committee (CEC)

The responsible investigator ensures that approval from an appropriately constituted Competent Ethics Committee (CEC) is sought for the clinical study. All changes in the research activity and all unanticipated problems involving risks to humans, including in case of planned or premature study end and the final report will be reported in a time frame of 12 months. Premature study end or interruption of the study is reported within 15 days. The regular end of the study is reported to the CEC within 90 days, the final study report will be submitted within one year after study end. Amendments are reported according to chapter 2.10.

2.4 Ethical Conduct of the Study

The study will be carried out in accordance to the protocol and with principles enunciated in the current version of the Declaration of Helsinki, the guidelines of Good Clinical Practice (GCP) issued by ICH[2,3], in case of medical device: the European Directive on medical devices 93/42/EEC and the ISO Norm 14155[4] and ISO 14971[22], the Swiss Law and Swiss regulatory authority's requirements. The CEC and regulatory authorities will receive an annual safety and interim reports and be informed about study stop/end in agreement with local requirements.

2.5 Declaration of interest

There is no conflict of interest (independence, intellectual, financial, proprietary, etc.)

2.6 Patient Information and Informed Consent

The investigators will explain to each participant the nature of the study, its purpose, the procedures involved, the expected duration, the potential risks and benefits, and any discomfort it may entail.
Each participant will be informed that the participation in the study is voluntary and that he/she may withdraw from the study at any time and that withdrawal of consent will not affect his/her subsequent medical assistance and treatment.
The participant will be informed that his/her medical records may be examined by authorized individuals other than their treating physician. All participants for the study will be provided a participant information sheet and a consent form describing the study and providing sufficient information for the participant to make an informed decision about their participation in the study. Patients will be given enough time to decide whether to participate or not in case of elective surgery (1-2 days), however in case of an emergency operation, the time frame will be shorter (1-2 h).
The study population includes vulnerable participants, we assume that at least 50% of all patients receiving a VPS belong to this vulnerable population. Without inclusion of these patients results of the study could not be adopted to the VPS patient population. In patients who are not capable of judgment (e.g. due to dementia, neurologic state (unconsciousness)), informed consent of the next of kin or an independent physician will be obtained. If the next of kin is not available, informed consent will be obtained from an independent physician on site. If at any time the patient shows signs that he is not willing to participate in the study he will not be included in the study. At a later time point, informed consent by the patient or his next of kin will be sought. Signs and symptoms showing that the participant is unwilling to participate in the study will result in the participant being excluded from participating. The patient information sheet and the consent form will be submitted to the CEC and to the competent authority (as applicable) to be reviewed and approved. The formal consent of a participant, using the approved consent form, will be obtained before the participant is submitted to any study procedure. The participant should read and consider the statement before signing and dating the informed consent form and will be given a copy of the signed document. The consent form will also be signed and dated by the investigator (or his designee) and it will be retained as part of the study records.

2.7 Participant privacy and confidentiality

The investigator affirms and upholds the principle of the participant's right to privacy and that they shall comply with applicable privacy laws. Especially, the anonymity of the participants shall be guaranteed when presenting the data at scientific meetings or publishing them in scientific journals.
Individual subject medical information obtained as a result of this study is considered confidential and disclosure to third parties is prohibited. Subject confidentiality will be further ensured by utilising subject identification code numbers to correspond to treatment data in the computer files.
For data verification purposes, authorised representatives of the Sponsor
(-Investigator), a competent authority (e.g. Swissmedic), or an ethics committee may require direct access to parts of the medical records relevant to the study, including participants' medical history.

2.8 Early termination of the study

The Sponsor-Investigator may terminate the study prematurely according to certain circumstances, for example:

- ethical concerns
- insufficient participant recruitment
- when the safety of the participants is doubtful or at risk, respectively
- alterations in accepted clinical practice that make the continuation of a clinical trial unwise
- early evidence of benefit or harm of the experimental intervention

2.9 Protocol amendments

Substantial amendments are only implemented after approval of the CEC and CA respectively.
Under emergency circumstances, deviations from the protocol to protect the rights, safety, and well-being of human subjects may proceed without prior approval of the sponsor and the CEC/CA. Such deviations shall be documented and reported to the sponsor and the CEC/CA as soon as possible.
All non-substantial amendments are communicated to the CA as soon as possible if applicable and to the CEC within the Annual Safety Report (ASR).

3. BACKGROUND AND RATIONALE

3.1 Background and Rationale

- VPS placement is one of the most frequent procedures in neurosurgical practice and is done approximatively once per week in our clinic (40 to 50 cases per year). The indications vary from normal pressure hydrocephalus in elderly patients to VPS-dependency after subarachnoid hemorrhage, infection or trauma, mostly in younger patients[5-7]. The position of the proximal ventricular catheter is important since it influences the possible malfunction of the VPS[8-11]. With freehand placement rates of malpositioned VPS are still high[12].

For the improvement of accuracy in proximal VPS placement, navigation-based insertion techniques have been developed. VPS placement using stereotactic navigation has shown a high accuracy of catheter placement, while the main limitations are that for referencing, the head of the patient needs to be fixed in a head holder and the preoperative set-up can be time-consuming[13]. US-G VPS placement using a burr hole probe was described as an alternate for image-guided VPS placement technique[14]. For US-G VPS placement head fixation or preoperative registration is not needed. Initially, the entry point is measured according to standard anatomical landmarks, thereafter the catheter is placed into the ventricle under real-time US guidance along the depicted trajectory[14]. To date, most of the clinical evidence regarding image-guided VPS placement derives from small retrospective cohort studies [15-17].

Installation and use of image guidance techniques add extra time to the standardized procedure of VPS placement, leading to longer operation times, and higher costs. Besides that patients will be anasesthesized for a longer time, leading to possible complications of prolonged anesthesia. An ideal image guidance technique should, therefore, be easy to handle, and not too time-consuming.

The rationale of this study is to prospectively compare US-G VPS placement to stereotactic navigation in a randomized controlled fashion with the surgical intervention time as a primary outcome.

3.2 Investigational Product (treatment, device) and Indication

- US navigation will be done by BK Medical 5000 US (bk medical Medizinische Systeme GmbH, Quickborn, Germany) using a burr hole probe (type 9063 N11C5S, 11-5 MHz). After placement of two horizontally overlapping burr holes with a 14-9mm high-speed drill at the Kocher point (creating a lying 8), real-time navigation will be done holding the US probe in the coronal plane. The instant inline trajectory will be used for placement of the ventricular catheter with the tip exactly at the foramen of Monro. The length of the catheter will be determined after visual placement at the foramen of Monro. A detailed technical note about the use of this US burr hole probe for EVD placement has been published in 2017[14].
- Stereotactic navigation will be done by Brainlab Dual Curve System (Brainlab AG, Munich, Germany) with cranial navigation software version 3.1. Preoperative planning of the exact location of the entry point and trajectory including the length of the ventricular catheter will be done the day before the operation (or in emergency operations directly preoperative). Placement of the catheter will be done using the navigated stylet.

3.3 Preclinical Evidence

N/A

3.4 Clinical Evidence to Date

There is to date no data available in the literature comparing surgical intervention time between US-G, and stereotactic navigated VPS placement.
Literature about the comparison of US-G and stereotactic navigated VPS placement is sparse. In one retrospective cohort study, US-G VPS placement has been compared to stereotactic navigation regarding the accuracy of catheter placement. No difference has been observed between these two navigation methods, whereas they were both better than freehand placement[15]. No prospective data is available for comparison.
Most of the clinical evidence regarding navigated VPS placement is from retrospective cohort studies or clinical notes[15-17]. Different studies showed that stereotactic- and US navigated ventricular catheter placement is significantly more accurate than freehand placement and that the use of these navigation techniques reduces proximal VPS failure[9,11,15-17,23,24].
Detailed use of the BK medical burr hole probe for real-time US-G EVD placement has been published in a technical note in 2017[14].

3.5 Dose Rationale / Medical Device: Rationale for the intended purpose in the study (pre-market MD)

The main aim of this study is to focus on the safety and feasibility of US-G VPS placement, with a special interest lying on the surgical intervention time (primary outcome). No preplanning, head fixation, or preoperative registration is needed. It is a real-time navigation method with direct visualization of the correct catheter placement.

US itself is not known to have any harmful side effects and does not contain harmful radiation, therefore it would be an optimal solution in the daily practice for routine use in all patients, especially in children and pregnant women.

3.6 Explanation for the choice of the comparator (or placebo)

Stereotactic navigated VPS placement is in use for many years. It has great accuracy even with slit ventricles[25]. During navigation, pre-planned images can be seen without real-time correction, as opposed to US guidance. Preplanning, head fixation, and preoperative registration are mandatory, possibly prolonging surgical intervention time and therefore leading to higher operating costs and longer duration of anesthesia.
No prospective data is available for comparison of US-G and stereotactic navigated VPS placement.

3.7 Benefits / Risks

<u>US navigation:</u> Compared to stereotactic navigation its use is fast with almost no extra time and extra cost, it does not need head fixation, preoperative planning nor referencing, thus shortening surgical intervention time. With real-time guidance, there is a correction for brain shift and new space-occupying lesions distorting the ventricular anatomy.
One of the main limitations of US is that a learning curve exists at first and that quality and understanding of US images can be limited, leading, at first, to longer surgical times. For placement of the burr hole probe on the cortex and insertion of the catheter with US guidance, the burr hole needs slight enlargement. This is a technical matter easily solved by a Kerrison punch, or by the placement of two overlapping burr holes resulting in a lying eight.
<u>Stereotactic navigation:</u> The method has great accuracy even in pediatric patients and patients with slit ventricles[25]. The patient's head needs to be fixed in a head clamp and the exact location of the entry point, the trajectory and the length of the catheter are pre-planned on the preoperative CT imaging. Fixation of the head with a head clamp holds risks for complications such as bleeding or infection. Planning and referencing of the navigation is time-consuming and prolongs the anesthesia time. The time factor is also a problem in an emergency operation. Since this is not a real-time navigation method, there is no correction for brain shift *e.g.* after placement of the burr hole (when air is entering the skull) or for new space-occupying lesions occurring in the time between the CT scan, and the operation.

3.8 Justification of choice of the study population

The study population will consist of patients above the age of 18 years receiving a VPS due to any reason. The study population includes vulnerable participants. In patients not capable of judgment (*e.g.* dementia/ neurologic state) informed consent of the next of kin will be obtained. Signs and symptoms showing that the participant is unwilling to participate in the study will result in the participant being excluded from participating.

4. STUDY OBJECTIVES

4.1 Overall Objective

- The main purpose of this study is to prospectively evaluate the feasibility of US navigation compared to stereotactic navigated VPS placement.

4.2 Primary Objective

To assess for the feasibility of the US navigation method we chose the surgical intervention time for comparison between the two navigation methods, which is a very well standardized time in minutes and is recorded for every operation. Any navigation method will probably prolong the time spent in the OR, but the time spent for US navigation will eventually be shorter than the time spent for stereotactic navigation, what would be very interesting in terms of cost efficiency, and burden for the patients (since additional anaesthesia time might be harmful).

4.3 Secondary Objectives

Further we are interested in special aspects of safety and feasibility of the navigation methods in direct comparison to each other, such as positioning of the catheter (optimal/not optimal, grade I to IV, safety), rate of VPS dysfunction and revision surgery (safety), number of ventricular puncture attempts that have been made (safety and feasibility), operation and anaesthesia times (feasibility), and any complications and morbidity (safety), as well as death (safety).

4.4 Safety Objectives

- Perioperative complications and mortality will be assessed for (see 4.3).

5. STUDY OUTCOMES

5.1 Primary Outcome

The primary outcome is the surgical intervention time. This is the time spent in the OR by the surgeon and includes the preparation time together (patient positioning, head clamping in the steretocatic navigation group) with the operation time of the neurosurgical part. Beginning ("Lagerung") and ending ("Naht neurosurgical part") of this time interval is clearly defined and will be in a standardized manner recorded by blinded anaesthesiologists. In the stereotactic navigation group, an additional 5 minutes will be added to the surgical intervention time for the preplanning of the navigation the day before the operation on the Brainlab workstation.

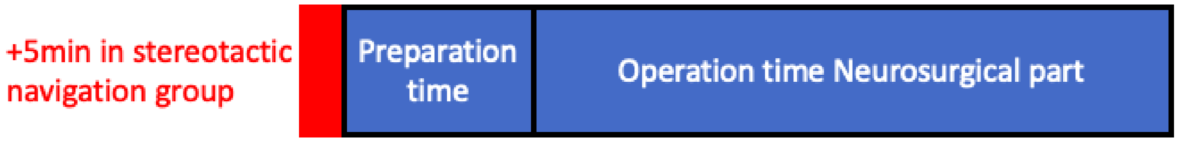

Figure 1: Composition of the surgical intervention time (preparation time plus operation time neurosurgical part), plus additional five minutes in the stereotactic navigation group.

5.2 Secondary Outcomes

- Secondary outcomes are:
  - Operation time in minutes (time in minutes from "Schnitt" to "Naht")
  - Anaesthesia time in minutes (time in minutes from "Beginn Anästhesie" to "Ende Anästhesie")
  - Number of puncture attempts
  - Catheter placement (optimal vs. not optimal): Optimal catheter placement has been defined as free-floating within the CSF without touching the ventricle wall or septum pellucidum, and the tip of the catheter located at the foramen of Monro showing an optimal length of the catheter. Catheters that did not fulfill all criteria have been defined as not optimal placed. Positioning of catheters will be further graded according to Yim et al. into grades I to IV (grade I: catheter terminates in the ipsilateral frontal horn, grade II: catheter terminates in contralateral frontal horn, grade III: catheter terminates in non-targeted CSF spaces, grade IV: catheter terminates intraparenchymally)[10].
  - Volumetry of side ventricles pre- and postoperatively in cm^3 (number and relative change)
  - Evan's Index[18] pre- and postoperatively (number and relative change)
  - Complications (infection, bleeding, complications ass. with navigation method), mortality
  - Revision surgery (yes/no) and reason for revision

5.3 Other Outcomes of Interest

N/A

5.4 Safety Outcomes

- The positioning of the catheter
- Number of ventricle puncture attempts
- VPS dysfunction, the revision rate
- perioperative complications (bleeding, infection), complications due to head clamp
- death, coma (<1% expected)

6. STUDY DESIGN

6.1 General study design and justification of design

- This is a prospective, randomized controlled study in a superiority fashion. The primary outcome (surgical intervention time) is well standardized and recorded by anaesthesiology for every operation. Some of the secondary outcomes are measured by a blinded neuroradiologist. The method of randomisation is 1:1. We chose this study design, in order to achieve evidence of the best quality while minimizing possible bias.

Patients will be randomized in two groups at admission in a 1:1 rate: US or stereotactic navigation group. Randomisation will be stratified for age (under 40 years vs. over 40 years). On the day of the elective operation, the VPS will be placed with the randomized navigation method. The patient is blinded for the navigation method, but there is no possibility to perform this study in a double-blinded fashion since the surgeon cannot be masked to the method. All patients in the stereotactic navigation group need to have a CT scan that is not older than 5 days counted backwards from the operation day. In the US navigation group there will be performed a new CT at admission if the old scan (CT or MRI) is older than 90 days. In patients allocated to stereotactic navigation the entry point, trajectory, and length of the catheter will be pre-planned the day before the operation on the 1 to 5 days old CT scan. The patients allocated to US navigation will not receive any pre-planning.

All adult (> 18 years) patients receiving a VPS in an elective, semi-elective, or urgent manner will be included in the study. These patients suffer from different forms of hydrocephalus (*e.g.* hydrocephalus internus (malresorptivus, occlusivus), hydrocephalus externus, hydrocephalus communicans, normal pressure hydrocephalus) due to different diseases, thus creating a heterogeneous population. For each group we plan to include 65 patients, giving a total of 130 + 6 = 136 patients and hence a study duration of 4 years.

The primary outcome (surgical intervention time) is recorded in a standardized manner by anasesthesiology for every operation in our department. In the stereotactic navigation group, 5 minutes will be added to the surgical intervention time accounting for navigation preplanning. Some of the secondary outcomes (catheter placement (optimal vs. not optimal, grade I to IV), volumetry of side ventricles, Evans' Index) will be analysed by a blinded neuroradiologist.

At admission, demographic and medical history will be obtained from the patients. Neurologic examination with analysis of GCS, modified Rankin Scale (mRS), Glasgow Outcome Scale (GOS) will be performed, and neurological symptoms (headaches, vomitus, gait disturbances, urinary incontinence, dementia, coma, delir) will be recorded. Patients in the US group need to have a CT scan that is not older than 90 days (old CT scans can be used if not older than 90 days, otherwise a new CT scan will be performed at atdmission), and patients in the stereotactic navigation group will receive a CT scan on the day of admission. From this CT scan, the Evans' index[18] will be measured and volumetry of the side ventricles will be done. In the stereotactic navigation group, the CT scan is used for pre-planning and referencing of the stereotactic navigation.

At day 0 (operation day) surgical intervention time, operation time and anaesthesia time, the number of puncture attempts, and peri-, as well as postoperative, complications will be assessed.

Postoperative during the hospital stay a CT scan will be performed (between 2nd and 5th day) for analysis of catheter positioning (optimal vs. not optimal, grade I to IV), volumetry of the side ventricles, and Evans' index. Neurologic examination with analysis of GCS, modified Rankin Scale (mRS), Glasgow Outcome Scale (GOS) will be performed, and neurological symptoms (headaches, vomitus, gait disturbances, urinary incontinence, dementia, coma, delir) will be recorded. Postoperative complications such as bleeding, rate of revision surgery and reason for revision, duration of hospital stay in days, duration of ICU stay in days, and peri-/postoperative complications will be assessed.

The first follow-up will take place 6-8 weeks postoperatively. Neurologic examination with analysis of GCS, modified Rankin Scale (mRS), Glasgow Outcome Scale (GOS) will be performed, and neurological symptoms (headaches, vomitus, gait disturbances, urinary incontinence, dementia, coma, delir) will be recorded. Assessment of postoperative complications (bleeding, complications due to head clamp, VPS infections, death), as well as type and reason for revision surgery, will be made.

The second follow-up will be performed 6 months postoperatively and includes a cCT scan (catheter position (optimal vs. not optimal, grade I to IV), calculation of Evans' index, volumetry of side ventricles), neurologic examination (analysis of GCS, modified Rankin Scale (mRS), Glasgow Outcome Scale (GOS), and neurological symptoms (headaches, vomitus, gait disturbances, urinary incontinence, dementia, coma, delir)), analysis of postoperative complications (bleeding, complications due to head clamp, VPS infections, death), and type and reason for revision surgery.

6.2 Methods of minimising bias

6.2.1 Randomisation

Randomisation will be performed by an independent individual using a stratified simple randomisation procedure as implemented in the electronic data capture software REDCap. An allocation ratio of 1:1 ensure a balance in sample size across both groups over time. The randomisation has been stratified according to age of the patients (under 40 years/over 40 years).

6.2.2 Blinding procedures

- Participants will be blinded for the navigation method they are randomized to. There is no possibility to blind the treating physician since he has to place the VPS with the selected navigation method and stereotactic navigation needs a pre-planning the day before the operation. The primary outcome is recorded by the anaesthesiology (not involved doctor), and some of the secondary outcomes will be measured by a blinded neuroradiologist who does not know the selected navigation method.

6.2.3 Other methods of minimising bias

- Prospective acquisition of data through CRF forms through an electronic software (REDCap).

6.3 Unblinding Procedures (Code break)

- Since the treating physicians are not blinded for the allocated intervention, there is no need for specific unblinding procedures.

7 STUDY POPULATION

7.1 Eligibility criteria

Participants fulfilling <u>all</u> of the following <u>inclusion</u> criteria are eligible for the study:

- Informed Consent as documented by signature (Appendix Informed Consent Form)
- Patients undergoing elective or emergent VPS placement
- frontal or occipital shunt placement
- Patients older than 18 years

The presence of any of the following <u>exclusion</u> criteria will lead to exclusion of the participant:

- Patients under the age of 18 years
- No informed consent.
- Emergency surgery if there is no time for installation of any navigation system
- Revision surgery using the same side and location, or revision surgery where no completely new shunt will be placed
- Ventriculoatrial and ventriculopleural placement
- Women who are pregnant or breast feeding
- Intention to become pregnant during the course of the study
- Enrolment of the investigator, his/her family members, employees, and other dependent persons

7.2 Recruitment and screening

Recruitment will take place either in the neurosurgical outpatient clinic or in the ER-Department of the University Hospital of Basel. The recruitment will be done by one of the clinics physicians who will be trained for this particular study. There will be no payment or compensation given to the participants.

7.3 Assignment to study groups

All patients entering the hospital for elective or emergent VPS surgery will be randomized in 1:1 fashion to one of the study groups at admission or the day before the operation. The randomisation process is further described in chapter 6.2.1. and will be performed by one of the trained physicians using the REDCap software.

7.4 Criteria for withdrawal/discontinuation of participants

Patients will be withdrawn from the study in case of withdrawal of informed consent or non-compliance, as well as patients with VPS dysfunction receiving revision surgery on the same side and location. If the surgeon shows non-adherence to the allocated navigation method (estimated in 1% of all cases), the patient will also be included, but will be included in the as treated analysis. Patients will not be replaced.

8 STUDY INTERVENTION

8.1 Identity of Investigational Products (treatment / medical device)

VPS placement by US-G or stereotactic navigation.

8.1.1 Experimental Intervention (treatment / medical device)

- US: navigation: BK Medical 5000 with burr hole probe (Type 9063 N11C5S, 11-5MHz)

8.1.2 Control Intervention (standard/routine/comparator treatment / medical device)

Stereotactic navigation: Brainlab Dual Curve System (cranial navigation software version 3.1.)

8.1.3 Packaging, Labelling, and Supply (re-supply)

- N/A

8.1.4 Storage Conditions

- N/A

8.2 Administration of experimental and control interventions

8.2.1 Experimental Intervention

At our institution, we routinely insert a (right) frontal VPS, while rarely an occipital VPS is placed. Since 2014, the abdominal shunt placement is performed by a visceral surgeon using the laparoscopic technique[26]. The "Kocher entry point" (measured at 11-12cm from Nasion and 3-3.5cm lateral to the midline) is used as the frontal entry point, the "Trigonum entry point" (measured at 4cm behind, and 5.5cm above the external auditory canal) is used as the occipital entry point. All catheters are connected to a burr-hole reservoir, a programmable valve (Codman Hakim®, Integra LifeSciences, USA), and a distal peritoneal catheter (Figure 2).

There is no pre-planning before starting the operation. After intubation, the head of the patient is placed on a horseshoe head holder slightly rotated to the contralateral side of the operation (Figure 3). There is no need to register the navigation.

Patients in the US cohort need to have a CT or MR scan not older than 90 days old (counted back from the operation day).

Directly before the operation, the distal shunt parts (peritoneal catheter, valve, and burrhole reservoir) are connected to each other and connections are saved with sutures.
After skin incision a large burr hole is placed with a 14-11mm high speed drill (Figure 4) at 12cm from Nasion and 3cm lateral to the intended side of shunt placement. Lateral enlargement of the burr hole is done until passing of the catheter through the adapted guide-channel lateral to the US burr hole probe is possible. US-G (BK Medical 5000, bk medical Medizinische Systeme GmbH, Quickborn, Germany) VPS placement is done using a burr hole probe (type 9063 N11C5S, 11-5 MHz) onto which a sterile single-use guide channel, adaptable for different catheter diameters is mounted (Figure 5 and 6).

Following dural opening, real-time US navigation is done holding the US probe in the coronal plane (Figure 7), showing a cross-section through both frontal horns. Saline irrigation is applied to achieve better contrast. The inline trajectory (Figure 7, in yellow) is used as guidance for positioning of the ventricular catheter with the tip at the foramen of Monro, then Foraman Monro and Choroid plexus are visualized, and the catheter is inserted. After positioning of the proximal catheter, under real-time visualisation (Figure 8), the US probe is removed, the catheter is shortened and connected to the burr-hole reservoir. The remaining procedure is similar to any other VPS placement, where the valve is connected to the burr hole device and the distal catheter, which is then implanted laparoscopically into the abdomen.

The physicians are already trained in the use of the US burr hole probe since it has been in use for many other operations, and VPS as well as EVD placement before. The operation will be performed by a trained neurosurgeon or by an advanced resident under supervision.

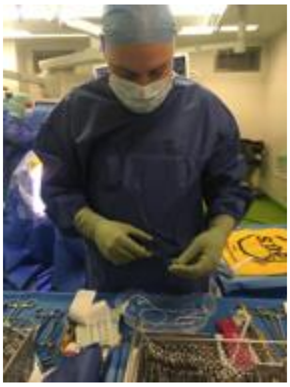
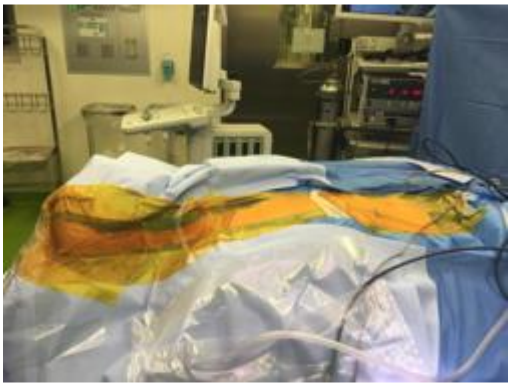
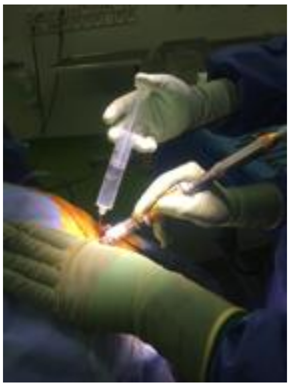

Figure 2: Connection of the valve and the peritoneal catheter to a large burr hole reservoir.
Figure 3: Positioning and covering of the patient for standard right frontal VPS placement (head left, abdomen right).
Figure 4: Placement of the burr hole, enlargement to the lateral side.

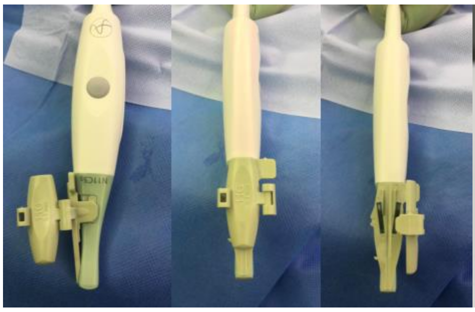
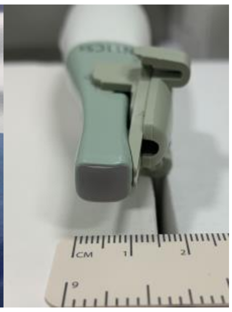
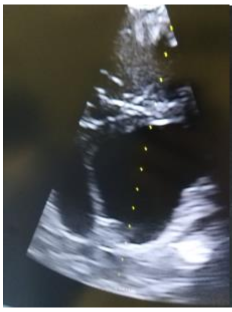
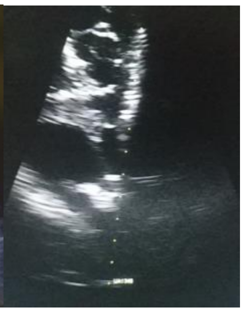

Figure 5: US burr hole probe attached to the guidance tool for the ventricular catheter, adaptable for the exact diameter of catheter (disposable material, single-use).
Figure 6: Image of the diameter of the US burr hole probe together with the attached guidance.
Figure 7: Real-time US navigation showing a coronal plane presenting both frontal horns of the lateral ventricles and the trajectory for catheter placement (in yellow).
Figure 8: Real-time US navigation showing a coronal plane presenting the catheter (hyperintense) entering the ventricle along the trajectory (in yellow).

8.2.2 Control Intervention

Patient in the stereotactic group need to have a CT scan that is not older than 5 days counted backwards from the operation day.
The exact location of the entry point, the trajectory, and the length of the catheter will be pre-planned on the newest CT at a Brainlab Workstation using cranial navigation software version 3.1. (Brainlab AG, Munich, Germany). After intubation the head of the patient will be fixed in a Mayfield head clamp slightly rotated to the contralateral side of the operation. Before starting the operation, the navigation will be registered. After that the incision will be marked at the pre-planned entry point. The distal shunt parts are also connected directly before the start of the operation. For placement the ventricular catheter will be inserted with the navigated stylet exactly along the planned trajectory. After functional control the catheter is cut in the precalculated length, and connected to the distal shunt parts. The other steps of the operation are exactly the same as described above for US-G VPS placement.

The physicians are already trained in using the Brainlab navigation since it is in use for almost every craniotomy and for biopsies. The operation is performed by a trained neurosurgeon or by an advanced resident under supervision.

8.3 Dose / Device modifications

- In case of very small ventricular width based on the surgeon's individual judgement one can decide to use stereotactic navigation instead of US navigation. Due to preoperative planning and necessity of a CT scan at admission this crossover has to be done preoperatively. Vice versa, there is the possibility to intraoperatively crossover from stereotactic navigation to US navigation in case of unsuccessful placement with stereotactic navigation (*e.g.* in cases with new mass effect compared to the preoperative CT scan and distorted ventricular anatomy).

8.4 Compliance with study intervention

During the perioperative period, compliance to the randomized navigation method will be monitored by study nurses. In cases of switched navigation method pre- or intraoperatively (see 8.3.) the reasons will be recorded and there will be an intention-to-treat analysis.

8.5 Data Collection and Follow-up for withdrawn participants

Withdrawn patients will be clinically followed up as non-participants, 6-8 weeks postoperatively in our outpatient clinic. A second follow-up will be performed 6 months postoperatively including a native CT scan.

8.6 Trial specific preventive measures

There are no specific restrictions associated with either navigation method. Patients sensitive to radiation (*e.g.* pregnant women or children) are excluded from the study (see eligibility criteria 7.1.) due to follow-up with CT scan. To our knowledge there are no contraindications for US of the brain, and no harmful side effects of US are described in the literature.

8.7 Concomitant Interventions (treatments)

- N/A

8.8 Study Drug / Medical Device Accountability

The devices for both of the navigation method (Brainlab stereotactic navigation system and BK 5000 medical US system) are stored in an equipment room in the OR tract of the university hospital of Basel. Brainlab planning workstation is located in the library of the university hospital of Basel.
All the equipment is reusable and partly sterile (*e.g.* US burr hole probe and reference star for the stereotactic navigation).

8.9 Return or Destruction of Study Drug / Medical Device

- Return or destruction of either medical device is according to standard procedures.

9 STUDY ASSESSMENTS

9.1 Study flow chart / table of study procedures and assessments

Study Periods	Screening Admission	Treatment, Intervention Period			Follow-up	
Visit	1	2	3	4	5	6
Time (hour, day, week)	1 day preop.	Opera-tion day	2-5 days post-op. (48-120h)	At discharge (approx. 7 days postop.)	6-8 weeks post-op.	6 months postop. (150-210 days postop.)
Patient Information and Informed Consent	x					
Randomization	x					
Demographics (age, sex)	x					
In- /Exclusion Criteria	x					
Neurologic examination	x		x	x	x	x
cCT scan	(x stereotactic navigation group, 1 to 5 days preoperative)		x (2nd to 5th day or earlier if neurologic symptoms)			x
Medical history	x					
Primary outcome (Surgical intervention time)		x				
Secondary Outcomes		x	x	x	x	x
VPS dysfunction		(x)	x	x	x	x
Operative Revision and reasons		(x)	x	x	x	x
Operation and anaesthesia time		x				
Number of puncture attempts		x				
Complications		x	x	x	x	x
Hospitalisation time (days)				x		
ICU time (days)				x		
**Discharge destination**				**x**		
Adverse events		x	x	x	x	x
**Death**		**x**	**x**	**x**	**x**	**x**

9.2. Assessments of outcomes

9.2.1. Assessment of primary outcome

Primary outcome (surgical intervention time) will be recorded for every operation by anaesthesiology, as it is standard in our department. This time spans the time spent in the OR by the neurosurgeon, beginning with any intervention on the patient (*e.g.* positioning, head clamping) and ends when the neurosurgical part of the operation is finished, so it consists of the preparation time, and the operation time of the neurosurgical part of the operation together. To minimize bias, the visceral surgery part of the operation (laparascopically assisted insertion of the peritoneal catheter intraperitoneally) will not be included into this time interval.

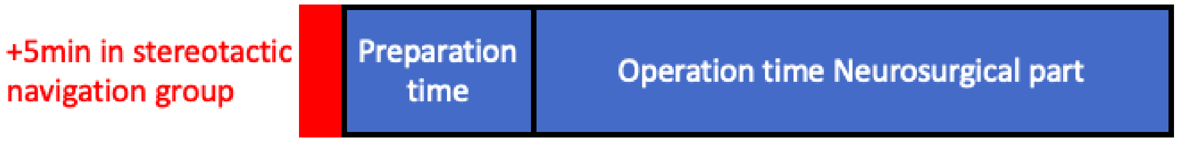

Figure 1: Composition of the surgical intervention time (preparation time plus operation time neurosurgical part), plus additional five minutes in the stereotactic navigation group.

9.2.2. Assessment of secondary outcomes

Operation time and anesthesia time in minutes: these times will be assessed at the operation day by anaesthesiology
Number of puncture attempts: this will be assessed at the operation day by the operating surgeon
Catheter placement (optimal vs. not optimal): between the 2nd and 5th postoperative day a CT scan will be made and VPS positioning will be assessed. Optimal catheter placement is defined as free-floating within the CSF without touching the ventricle wall or septum pellucidum, and the tip of the catheter located at the foramen of Monro showing an optimal length of the catheter. Catheters that did not fulfil all criteria have been defined as not optimal placed. Positioning of catheters will be further graded according to Yim et al. into grades I to IV (grade I: catheter terminates in the ipsilateral frontal horn, grade II: catheter terminates in contralateral frontal horn, grade III: catheter terminates in non-targeted CSF spaces, grade IV: catheter terminates intraparenchymally)[10].
The cCT scan will be repeated during second follow-up after 6 months. This outcome will be assessed by partially blinded neuroradiologists.
Volumetry of side ventricles pre- and postoperatively in cm^3 (number and relative change): This will be assessed on the pre- and postoperative CT scans by partially blinded neuroradiologists.
Evan's Index pre- and postoperatively (number and relative change): This will be assessed on the pre- and postoperative CT scans by partially blinded neuroradiologists.
Complications (infection, bleeding, complications ass. with navigation method), mortality: rate and detail of complications will be assessed directly postoperative during the whole hospitalisation time and during the 1st and 2nd follow-up by the study nurse, the same accounts for mortality (including the reason for death if known).
Revision surgery (yes/no) and reason for revision: the rate of revision surgery as well as the indication for revision will be reported by the study nurse.
VPS dysfunction: the rate of VPS dysfunction will be assessed directly postoperative during the whole hospitalisation time, and during the 1st and 2nd follow-up by the study nurse.

9.2.3 Assessment of other outcomes of interest

- N/A

9.2.4 Assessment of safety outcomes

9.2.4.1 Adverse events

- Adverse events in our study mean death due to any reason (related or unrelated to the study) and any complications referable to any of the navigation methods (*e.g.* headholder complications for the stereotactic navigation method, local bleeding at the burr hole for the US navigation method (due to enlarged burr hole).

9.2.4.2 Laboratory parameters

There will be no laboratory parameters analysed in this study.

9.2.4.3 Vital signs

There will be no vital signs analysed in this study.

9.2.5 Assessments in participants who prematurely stop the study

No further assessments will be performed for such patients.

9.3 Procedures at each visit

9.3.1 Visit 1: Screening and admission

Randomisation, demographics (age, gender), height, weight, body mass index (BMI), neurologic examination (GCS, modified Rankin Scale (mRS), Glasgow Outcome Scale (GOS), neurological symptoms (headaches, vomitus, coma, gait disturbances, dementia, urinary incontinence, motor deficit, sensory deficit, aphasia, delir, others), medical history (underlying disease causing hydrocephalus (NPH, SAH, IVH, other type of bleeding, trauma, tumor, congenital, other), prior EVD, prior VPS, or prior head operations including details), cCT scan (US group not older than 90 days, stereotactic navigation group not older than 5 days, measurements: Evans' index, volumetry of side ventricles). Randomisation and cCT scan (indications see above) is the only measurements done for the study, all the other measurements are generally assessed for all patients receiving this operation.

9.3.2 Visit 2: Operation day

Primary outcome (surgical intervention time), shunt side (right, left), shunt location (frontal, occipital), ventricular catheter length, number of surgeons, experience of main surgeon, shunt/valve manufacturer, type of valve (adjustable, non-adjustable), valve pressure, number of accessory incisions, shunt dysfunction, revisions surgery, indication for revision, secondary outcomes (operation time, anaesthesia time, number of puncture attempts, complications including complication details), death including reason of death.
Number of surgeons, experience of main surgeon, number of accessory incisions, number of puncture attempts are the only measurements done for the study, all the other measurements are generally assessed for all patients receiving this operation.

9.3.3 Visit 3: 2nd to 5th postoperative day (48-120h postoperative)

Neurologic examination (GCS, modified Rankin Scale (mRS), Glasgow Outcome Scale (GOS), neurological symptoms (headaches, vomitus, coma, gait disturbances, dementia, urinary incontinence, motor deficit, sensory deficit, aphasia, delir), neurology better, headaches better, vomitus better, gait ataxia better, dementia better, urinary incontinence better), cCT scan (catheter position (optimal vs. not optimal, grade I to IV), Evans' index, volumetry of side ventricles, Evans' Index improvement, ventricle width reduction), shunt dysfunction including details (proximal/distal obstruction, proximal/distal dislocation, abdominal cause, dysfunction due to infection, disconnection, other), revision surgery and indication for revision (bleeding, infection, obstruction, misplacement, disconnection, proximal/distal dislocation, other), complications other than dysfunction (infection, blee,ding, seizure, fracture, other), death including reason of death.
No extra measurements are assessed for the study, all measurements are generally assessed for all patients receiving this operation.

9.3.4 Visit 4: Discharge (approx. 7 days postoperatively)

Neurologic examination (GCS, modified Rankin Scale (mRS), Glasgow Outcome Scale (GOS), neurological symptoms (headaches, vomitus, coma, gait disturbances, dementia, urinary incontinence, motor deficit, sensory deficit, aphasia, delir), neurology better, headaches better, vomitus better, gait ataxia better, dementia better, urinary incontinence better), shunt dysfunction including details (proximal/distal obstruction, proximal/distal dislocation, abdominal cause, dysfunction due to infection, disconnection, other), revision surgery and indication for revision (bleeding, infection, obstruction, misplacement, disconnection, proximal/distal dislocation, other), complications other than dysfunction (infection, blee,ding, seizure, fracture, other), duration of (postoperative) hospitalisation in days, duration of ICU stay in days, discharge destination (home, rehabilitation, nursing facility, other hospital, other), death including reason of death.
Duration of (postoperative) hospitalisation, and duration of ICU stay in days are the only measurements done for the study, all the other measurements are generally assessed for all patients receiving this operation.

9.3.5 Visit 5: 1st Follow-up (6-8 weeks postoperatively)

Time of follow-up (date and number of days postoperative), neurologic examination (GCS, modified Rankin Scale (mRS), Glasgow Outcome Scale (GOS), neurological symptoms (headaches, vomitus, coma, gait disturbances, dementia, urinary incontinence, motor deficit, sensory deficit, aphasia, delir), neurology better, headaches better, vomitus better, gait ataxia better, dementia better, urinary incontinence better), shunt dysfunction including details (proximal/distal obstruction, proximal/distal dislocation, abdominal cause, dysfunction due to infection, disconnection, other), revision surgery and indication for revision (bleeding, infection, obstruction, misplacement, disconnection, proximal/distal dislocation, other), complications other than dysfunction (infection, blee,ding, seizure, fracture, other), death including reason of death.
Number of days postoperative, neurologic examination, and neurologic symptoms are the only measurements done for the study, all the other measurements are assessed in a standard manner for all patients receiving this operation.

9.3.5 Visit 6: 2nd Follow-up (6 months postoperatively)

Time of follow-up (date and number of days postoperative), neurologic examination (GCS, modified Rankin Scale (mRS), Glasgow Outcome Scale (GOS), neurological symptoms (headaches, vomitus, coma, gait disturbances, dementia, urinary incontinence, motor deficit, sensory deficit, aphasia, delir), neurology better, headaches better, vomitus better, gait ataxia better, dementia better, urinary incontinence better), cCT scan (catheter position (optimal vs. not optimal, grade I to IV), Evans' index, volumetry of side ventricles, Evans' Index improvement, ventricle width reduction), shunt dysfunction including details (proximal/distal obstruction, proximal/distal dislocation, abdominal cause, dysfunction due to infection, disconnection, other), revision surgery and indication for revision (bleeding, infection, obstruction, misplacement, disconnection, proximal/distal dislocation, other), complications other than dysfunction (infection, blee,ding, seizure, fracture, other), death including reason of death.
Number of days postoperative, neurologic examination, and neurologic symptoms are the only measurements done for the study, all the other measurements are assessed in a standard manner for all patients receiving this operation.

10 SAFETY

10.3 Medical Device Category A studies

Device deficiencies and all adverse events (AE) including all serious adverse events (SAE) are collected, fully investigated and documented in the source document and appropriate case report form (CRF) during the entire study period, i.e. from patient's informed consent until the last protocol-specific procedure, including a safety follow-up period. Documentation includes dates of event, treatment, resolution, assessment of seriousness and causal relationship to device and/or study procedure [ISO 14155, 6.4.1.[4]].
For this study these are especially perioperative complications of VPS implantation and perioperative complications of either navigation method (*e.g.* headholder complications such as bleeding or infection for the stereotactic navigation and local bleeding at the burr hole due to enlargement for the US navigation).

10.3.1 Definition and Assessment of safety related events

Adverse Event (AE)

Any untoward medical occurrence, unintended disease or injury or any untoward clinical signs (including an abnormal laboratory finding) in participants, users or other persons whether or not related to the investigational medical device [ISO 14155: 3.2[4]].

Complications related to the operation and either of the navigation method will be reported and patient will receive the appropriate monitoring and, if necessary, treatment.

Adverse Device Effect (ADE)

Adverse event related to the use of an investigational medical device [ISO 14155: 3.1[4]].

Serious Adverse Event (SAE) [European regulation on medical devices 2017/745, art. 58[27]]. Any adverse event that led to any of the following:

(a) death,
(b) serious deterioration in the health of the subject that resulted in any of the following:

(i) life-threatening illness or injury,
(ii) permanent impairment of a body structure or a body function,
(iii) hospitalisation or prolongation of patient hospitalisation,
(iv) medical or surgical intervention to prevent life-threatening illness or injury or permanent impairment to a body structure or a body function,
(v) chronic disease,

(c) foetal distress, foetal death or a congenital physical or mental impairment or birth defect.

Device deficiency

Inadequacy of a medical device related to its identity, quality, durability, reliability, safety or performance, such as malfunction, misuse or use error and inadequate labelling [ISO 14155: 3.15[4]].

Health hazards that require measures

Findings in the trial that may affect the safety of study participants and, which require preventive or corrective measures intended to protect the health and safety of study participants SAE [ClinO Art. 37[19]].

Causal Relationship of SAE [MEDDEV 2.7/3 revision 3, May 2015[28]].

A causal relationship towards the medical device or study procedure should be rated as follows:

 Not related: The relationship to the device or procedures can be excluded.

 Unlikely: The relationship with the use of the device seems not relevant and/or the event can

be reasonably explained by another cause, but additional information may be obtained.

 Possible: The relationship with the use of the investigational device is weak but cannot be

ruled out completely. Alternative causes are also possible.

 Probable: The relationship with the use of the investigational device seems relevant and/or

the event cannot reasonably explained by another cause.

 Causal relationship: The serious event is associated with the investigational device or with

procedures beyond reasonable doubt.

Device deficiencies that might have led to an SAE are always related to the medical device.

10.3.2 Reporting of Safety related events

Important note concerning all following sections of this chapter 10.3.2: add, respectively adapt to other local requirements in case of international studies.

Reporting to Sponsor-Investigator:

Health hazard that require measures are reported to the Sponsor-Investigator within 24 hours upon becoming aware of the event:

Pregnancies

Pregnancies are an exclusion criteria for this study since the follow-up is performed with CT scans. All pregnancies are reported within 24 hours to the Sponsor-Investigator and patients are withdrawn from the study. Depending on the stage of the study and thus to the radiation dose, the outcome of the pregnancy shoud be followed.

Reporting to Authorities:

In Category A studies, the sponsor is subject to the notification requirements specified in Art. 15 of the MedDO of 17 October 2011 (SR 812.213)[21].

It is the Investigator's responsibility to report to the Ethics Committee via BASEC device deficiencies that could have led to serious adverse events if suitable action had not been taken, intervention had not been made, or circumstances had been less fortunate within 7 days [ClinO Art. 42[19]].

Health hazards that require measures are reported to the Ethics Committee via BASEC within 2 days [ClinO Art. 37[19]].

Periodic safety reporting:
A yearly safety update-report is submitted by the Investigator to the Ethics Committee via BASEC.

A report is submitted to Swissmedic by the Sponsor-Investigator, as defined in Art. 15a,b of the MedDO of 17 October 2011 (SR 812.213)[21].

11 STATISTICAL METHODS

11.1 Hypothesis

The hypothesis of the study is that patients undergoing VPS placement using US navigation have a shorter surgical intervention time than patients undergoing VPS placement using stereotactic navigation.

11.2 Determination of Sample Size

The sample size was estimated with the aim of showing a surgical intervention time reduction of 15 minutes (min) in the intervention arm compared to the control arm. The significance level was chosen to be 5%, while the power was chosen to be (1-β) = 80%.

Based on unpublished data from a pilot study assessing the surgical intervention time in patients undergoing VPS placement using US navigation, we calculated a mean surgical intervention time of 63 min with a standard deviation (SD) of 28.65 min. Because of the additional steps involved using stereotactic navigated surgery we assume an increased surgical invention time in those patients. Based on these assumptions we calculated various scenarios presuming different differences in surgical intervention time between the two arms (see Figure 9).

For our hypothesis for the sample size calculation, we then conservatively selected a scenario with a relatively modest difference between groups. This resulted in 58 patients with VPS placement surgery per group. Anticipating a 1% drop-out by deaths and a surgeons' non-adherence rate (change from control to treatment arm) of 5%, a total sample size of 130 + 6 = 136 patients (68 per group) is required (see Figure 9).

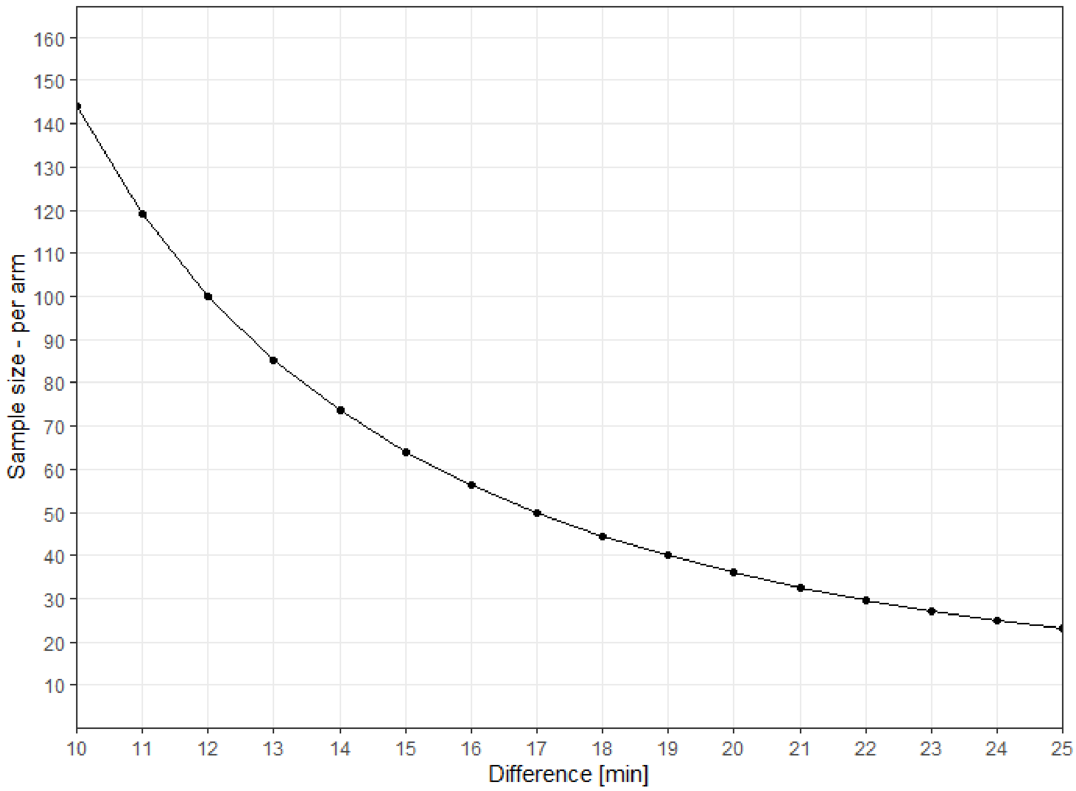

Figure 9: Sample size for different scenarios with 80% power and α = 0.05. The calculation is based on a standard deviation in surgical intervention time of 28.65 min with an anticipated drop-out rate of 1% and a non-compliance rate (change of treatment arm) of 5%.

11.3 Statistical criteria of termination of the trial

As no interim analysis is planned, no statistical criteria have been defined for early termination of the trial.

11.4 Planned Analyses

All analyses will be conducted using the statistical software package R[29]. Analyses will follow CONSORT guidelines[30] and intention-to-treat principles. A flowchart will describe the inclusion and follow-up of participants by study arm. Baseline characteristics will be described by study arm with summary statistics such as median and interquartile range or number and percentage; no formal testing between arms will be performed[31]. Outcomes will be described by arm using summary statistics.

11.4.1 Datasets to be analysed, analysis populations

The full analysis set (FAS) will include all patients that were randomized. All statistical analyses will be performed on the FAS according to the intention-to-treat principle (i.e. all participants will be analysed on the basis of the intervention to which they were randomly allocated).
The per-protocol (PP) set will include all participants in the FAS who fulfilled the eligibility criteria, for whom the surgery was completed as planned in the study protocol, and for whom the measurement of the primary outcome is available.

11.4.2 Primary Analysis

The primary outcome, surgical intervention time, will be assessed using a linear regression model, reporting adjusted mean differences between arms. The estimates will be reported with 95% confidence intervals (CI). The model will be adjusted for the most important risk factors. Further details will be provided in the statistical analysis plan.

11.4.3 Secondary Analyses

The secondary outcomes will be analysed using linear regression models (for the outcomes operation time, anaesthesia time, volumetry of side ventricles, and Evan's Index), logistic regression models (for the outcomes optimal catheter placement, complications, mortality and revision surgery), or a Poisson regression model (for the outcome number of puncture attempts).
All estimates will be reported with 95% CI. All models will be adjusted for the most important risk factors. We will compare each endpoint between the intervention and control arms. Further details will be provided in the statistical analysis plan.

11.4.4 Interim analyses

There is no interim analysis planned for this study.

11.4.5 Safety analysis

Safety will be assessed via a rigorous and detailed examination of adverse events between the treatment group and the control group. Safety endpoints will be assessed using the FAS.

Safety endpoints are the following events: positioning of the catheter, number of ventricle puncture attempts, VPS dysfunction, revision rate, perioperative complications (bleeding, infection), complications due to head clamp, death, and coma.

Binary safety endpoints will be compared as number of events between the two groups (US and stereotactic navigation) using a logistic regression. To compare the number of ventricle puncture attempts per patient between the treatment and control arm we will use a Poisson regression model.

11.4.6 Deviation(s) from the original statistical plan

If substantial deviations of the analysis as outlined in these sections are needed for whatever reason, the protocol will be amended. All deviations of the analysis from the protocol or from the detailed analysis plan will be listed and justified in a separate section of the final statistical report.

11.5 Handling of missing data and drop-outs

Missing baseline and outcome data will be summarized by study arm. As outlined above, the primary analyses will be the intent-to-treat population. In the case of missing data or drop-outs, we may adjust for further baseline variables which are associated with missing outcome data[32] (which is analogous to performing multiple imputation in the case of a single endpoint). We may consider multiple imputation as sensitivity analyses if necessary.

12 QUALITY ASSURANCE AND CONTROL

12.1 Data handling and record keeping / archiving

- All data will be encoded collected, stored and evaluated.
- A number for the screening period will be allocated to each participant. After electronic enrolment to the study, the participant will be assigned with a personal study number (e.g. 001, 002 etc.). All data collected for the study will be entered under the encoded study number only.
- Participant`s name, address, date of birth, screening- and study number will be documented in a written register ("patient identification list"). The patient identification list will be stored in the research study office of the department of Neurosurgery, University Hospital of Basel.

For statistical reasons age and gender will be collected in order to allow a comparison between the study arm and the control arm.
Access to the data will be provided to the Sponsor, the PI and Co-Investigators, the study nurse, and the statistician from CEB, University Hospital of Basel.

12.1.1 Case Report Forms

For each subject included in this study, a Case Report Forms (CRF) will be completed, dated and signed by the study investigator. A list with signatures and initials of all authorized persons will be filed in the trial master file (TMF).
Data will be recorded in the CRF from the source documents which may include medical notes, drug prescription charts (paper or electronic) and computerised laboratory reporting systems.
All data entered into the CRF will be also available in the individual participant file, either as print-outs or as notes taken by the investigator.
All requested information in the CRF will be completed in a neat legible manner. Data that is not available or not done will be made clear by adding "NA" or "ND". Possible corrections will be made in a way that does not obscure the original entry. Each CRF will be kept current to reflect the participant status at each phase during the course of the study.
Copies of protocols CRFs, originals of test result reports, drug dispensing logs, correspondence, records of informed consent and other documents pertaining to the conduct of the study will be kept on file by the investigator for a period of time specified by local law for the preservation of hospital patient documents. If a subject withdraws from the study, the reason will be noted on the CRF.
All participants receive a unique identification number (patient ID) and no person identifying data such as name or initials are collected in the CRF.

12.1.2 Specification of source documents

The investigator will maintain source documents for each patient included in the study, consisting of the paper CRF forms, case and visit notes (hospital or clinical medical records) containing demographic and medical information, laboratory data, electrocardiograms, and the results of any other tests or assessments.
Study related not encoded documents are stored as paper source data. These files will be stored in the research office of the department of Neurosurgery at the University Hospital of Basel. Only members of the study team will have access to this office.

12.1.3 Record keeping / archiving

All study data, including CRFs, TMF and informed consent forms will be archived for a minimum of 10 years after study termination or premature termination of the clinical trial. The study data will be archived in the research office of the Department of Neurosurgery University Hospital of Basel.

12.2 Data management

The study data recorded in the CRF will be transferred to a corresponding electronic CRF (e-CRF) by authorized persons (see Staff List).
The Principal Investigator and Co-Investigator at the study site will be responsible for assuring that the data entered into the e-CRF is complete, accurate, and that entry and updates are performed in a timely manner.
All information recorded in the e-CRFs will be traceable to the source documents in the patient's file and in the data source files.

12.2.1 Data Management System

The e-CRF will be implemented using the EDC software REDCap. The EDC software runs on a server maintained by the IT-department at University Hospital Basel.

12.2.2 Data security, access and back-up

REDCap data capture is accessible via a standard browser on a www-connected device.
Password protection and user-right management ensures that only authorized persons can enter the EDC system to view, add or modify data according to their permissions.

An integrated audit trail system maintains a record of initial entries and changes (reason for change, date and time of change and user identification). The database is backed up regularly according to the processes of the IT-department at University Hospital Basel.

12.2.3 Analysis and archiving

The EDC system will be locked after all CRF data was transferred, monitored and all raised queries have been resolved. The complete study dataset is exported from the database and transferred to the study statistician as well as the principal investigator through a secured channel. The exported data will be archived by the principal investigator.

12.2.4 Electronic and central data validation

Data entered into the e-CRF will be validated for completeness and discrepancies automatically. The data will be reviewed by the responsible investigator as well as an independent monitor. The monitor will raise queries using the query management system implemented in REDCap. Designated investigators have to respond to the query and confirm or correct the corresponding data. Thereafter the monitor can close the query.

12.3 Monitoring

The e-CRF and source data will be reviewed for completeness and accuracy through regular monitoring provided by the study site.
The study staff will be available for the monitoring visits in order to give access to the study files and give any kind of support needed.
The service will be provided by Julia Manzetti, clinical monitor from the Departement Klinische Forschung of the University Hospital of Basel.

12.4 Audits and Inspections

Inspections by regulatory authorities during the study or after the study is completed are performed to ensure proper study conduct and data handling procedures according to ICH-GCP[2,3] guidelines and regulatory requirements. Inspections may include verification of all source documents, e-CRF, site files, and a visual inspection of the study site. Direct access to all documents and sites involved in the study will be provided by the study staff members. In case of an announced inspection immediate notification of the other party is necessary.

12.5 Confidentiality, Data Protection

Direct access to source documents will be permitted for purposes of monitoring (12.3). The participants name or other personal identifiable data are not recorded in the CRF as well as eCRF. Subjects' confidentiality will be ensured by utilising unique identification numbers to correspond the data. Each participant will be coded by a number during the screening. After electronic enrolment to the study, the participant will be assigned with a unique personal study number (patient ID). All data collected for the study will be entered under the patient ID only.
The relation between the patient ID and participant`s name, address, date of birth and screening number will be documented in a written register ("patient identification list"). The patient identification list will be stored in the research study office of the department of Neurosurgery, University Hospital of Basel.
The Sponsor, PI, Co-Investigator and study nurses will have access to the protocol and dataset. While the CEB statistician will have access to the statistical codes during and after the study.
Study data entered into the EDC system is only accessible by authorized persons. Once the data of all subjects is transferred to the EDC system, the database will be locked and closed for further data entry. The complete study dataset is exported, encrypted and transferred to the principal investigator through a secured channel by the responsible Data Manager at University Hospital Basel.

12.6 Storage of biological material and related health data

After completion of the study the data and materials will be stored at REDCap data capture program which is accessible via a standard browser on a www-connected device. Password protection and user-right management will still apply and ensures that only authorized persons can enter the EDC system to view data and use it for further research according to their permissions.
Exported data will be archived by the principal investigator in a locked cupboard within the offices of the Neurosurgical Department of the University Hospital of Basel.

13 PUBLICATION AND DISSEMINATION POLICY

- The trial is registered at clinicaltrials.gov and at www.kofam.ch.
- The study protocol is submitted as publication in a peer reviewed medical journal for trial protocols (Trials).
- Publication of the final study results will be published in a top tier peer reviewed medical journal.

14 FUNDING AND SUPPORT

14.1 Funding

The study will be financially supported by the research foundation of the department of Neurosurgery of the University Hospital of Basel and we applied for further funding at different other foundations.

14.2 Other Support

N/A

15 INSURANCE

Insurance will be covered by the business liability insurance of the Department Neurosurgery, University Hospital of Basel.

APPENDICES

1. Medical Devices:
    a. US burr hole probe:
        - https://www.bkmedical.com/transducers/n11c5s-burr-hole/
    b. Brainlab Dual Curve System (cranial navigation software version 3.1.):
        - https://www.brainlab.com/de/chirurgie-produkte/uebersicht-ueber-neurochirurgie-produkte/kraniale-navigation/
2. List of involved staff members at University Hospital of Basel
3. Case Report Form (CRF)
4. Patient Information and Informed Consent
    a. For patients: prospective and retrospective inclusion
    b. For relatives: prospective and retrospective inclusion

REFERENCES

1. Declaration of Helsinki, Version October 2013, http://www.wma.net/en/30publications/10policies/b3/index.html. In.
2. International Conference on Harmonization (ICH, 1996) E6 Guideline for Good Clinical Practice. (http://www.ich.org/fileadmin/Public_Web_Site/ICH_Products/Guidelines/Efficacy/E6/E6_R2__Step_4.pdf). In.
3. International Conference on Harmonization (ICH, 1997) E8 Guideline: General Considerations for Clinical Trials http://www.ich.org/fileadmin/Public_Web_Site/ICH_Products/Guidelines/Efficacy/E8/Step4/E8_Guideline.pdf). In.
4. ISO 14155:2011 Clinical investigation of medical devices for human subjects -- Good clinical practice (www.iso.org) In.
5. Brean A, Eide PK. Prevalence of probable idiopathic normal pressure hydrocephalus in a Norwegian population. *Acta Neurol Scand.* 2008;118(1):48-53.
6. Hoh BL, Kleinhenz DT, Chi YY, Mocco J, Barker FG. Incidence of ventricular shunt placement for hydrocephalus with clipping versus coiling for ruptured and unruptured cerebral aneurysms in the Nationwide Inpatient Sample database: 2002 to 2007. *World Neurosurg.* 2011;76(6):548-554.
7. Little AS, Zabramski JM, Peterson M, et al. Ventriculoperitoneal shunting after aneurysmal subarachnoid hemorrhage: analysis of the indications, complications, and outcome with a focus on patients with borderline ventriculomegaly. *Neurosurgery.* 2008;62(3):618-627; discussion 618-627.
8. Janson CG, Romanova LG, Rudser KD, Haines SJ. Improvement in clinical outcomes following optimal targeting of brain ventricular catheters with intraoperative imaging. *J Neurosurg.* 2014;120(3):684-696.
9. Sampath R, Wadhwa R, Tawfik T, Nanda A, Guthikonda B. Stereotactic placement of ventricular catheters: does it affect proximal malfunction rates? *Stereotact Funct Neurosurg.* 2012;90(2):97-103.
10. Yim B, Reid Gooch M, Dalfino JC, Adamo MA, Kenning TJ. Optimizing ventriculoperitoneal shunt placement in the treatment of idiopathic intracranial hypertension: an analysis of neuroendoscopy, frameless stereotaxy, and intraoperative CT. *Neurosurg Focus.* 2016;40(3):E12.
11. Hayhurst C, Beems T, Jenkinson MD, et al. Effect of electromagnetic-navigated shunt placement on failure rates: a prospective multicenter study. *J Neurosurg.* 2010;113(6):1273-1278.
12. Kamenova M, Rychen J, Guzman R, Mariani L, Soleman J. Yield of early postoperative computed tomography after frontal ventriculoperitoneal shunt placement. *PLoS One.* 2018;13(6):e0198752.
13. Reig AS, Stevenson CB, Tulipan NB. CT-based, fiducial-free frameless stereotaxy for difficult ventriculoperitoneal shunt insertion: experience in 26 consecutive patients. *Stereotact Funct Neurosurg.* 2010;88(2):75-80.
14. Manfield JH, Yu KKH. Real-time ultrasound-guided external ventricular drain placement: technical note. *Neurosurg Focus.* 2017;43(5):E5.
15. Wilson TJ, Stetler WR, Al-Holou WN, Sullivan SE. Comparison of the accuracy of ventricular catheter placement using freehand placement, ultrasonic guidance, and stereotactic neuronavigation. *J Neurosurg.* 2013;119(1):66-70.
16. Kullmann M, Khachatryan M, Schuhmann MU. Ultrasound-guided placement of ventricular catheters in first-time pediatric VP shunt surgery. *Childs Nerv Syst.* 2018;34(3):465-471.
17. Crowley RW, Dumont AS, Asthagiri AR, et al. Intraoperative ultrasound guidance for the placement of permanent ventricular cerebrospinal fluid shunt catheters: a single-center historical cohort study. *World Neurosurg.* 2014;81(2):397-403.
18. Evans WJ. An encephalographic ratio for estimating ventricular enlargement and cerebral atrophy. *Archives of Neurology & Psychiatry.* 1942;47:931-937.
19. Verordnung über klinische Versuche in der Humanforschung (Verordnung über klinische

Versuche, KlinV) vom 20. September 2013 / Ordonnance sur les essais cliniques dans le cadre de la recherche sur l'être humain (Ordonnance sur les essais cliniques, OClin) du 20 septembre 2013. Ordinanza sulle sperimentazioni cliniche nella ricerca umana (Ordinanza sulle sperimentazioni cliniche, OSRUm) del 20 settembre 2013 In.

20. Humanforschungsgesetz, HFG Bundesgesetz über die Forschung am Menschen (Bundesgesetz über die Forschung am Menschen, HFG) vom 30. September 2011/ Loi fédérale relative à la recherche sur l'être humain (loi relative à la recherche sur l'être humain, LRH) du 30 septembre 2011 / Legge federale concernente la ricerca sull'essere umano (Legge sulla ricerca umana, LRUm) del 30 settembre 2011 In.

21. Medizinprodukteverordnung (MepV) vom 17. Oktober 2001 / Ordonnance sur les dispositifs médicaux (ODim) du 17 octobre 2001 / Ordinanza relativa ai dispositivi medici (ODmed) del 17 ottobre 2001. In.

22. DIN EN ISO 14971:2013-04, Medizinprodukte – Anwendung des Risikomanagements auf Medizinprodukte (ISO 14971:2007, korrigierte Fassung 1. Oktober 2007); Deutsche Fassung EN ISO 14971:2012. In.

23. Gilard V, Magne N, Gerardin E, et al. Comparison of electromagnetic neuronavigation system and free-hand method for ventricular catheter placement in internal shunt. *Clin Neurol Neurosurg.* 2017;158:93-97.

24. Jung N, Kim D. Effect of electromagnetic navigated ventriculoperitoneal shunt placement on failure rates. *J Korean Neurosurg Soc.* 2013;53(3):150-154.

25. Clark S, Sangra M, Hayhurst C, et al. The use of noninvasive electromagnetic neuronavigation for slit ventricle syndrome and complex hydrocephalus in a pediatric population. *J Neurosurg Pediatr.* 2008;2(6):430-434.

26. Schucht P, Banz V, Trochsler M, et al. Laparoscopically assisted ventriculoperitoneal shunt placement: a prospective randomized controlled trial. *J Neurosurg.* 2015;122(5):1058-1067.

27. European regulation on medical devices 2017/745. In.

28. MEDDEV 2.7/3 revision 3, May 2015. In.

29. 2018 RCT. R: A Language and Environment for Statistical Computing. . In. Vienna, Austria: R Foundation for Statistical Computing; 2018.

30. Schulz KF, Altman DG, Moher D, Group C. CONSORT 2010 statement: updated guidelines for reporting parallel group randomized trials. *Ann Intern Med.* 2010;152(11):726-732.

31. Pocock SJ, Assmann SE, Enos LE, Kasten LE. Subgroup analysis, covariate adjustment and baseline comparisons in clinical trial reporting: current practice and problems. *Stat Med.* 2002;21(19):2917-2930.

32. Sullivan TR, White IR, Salter AB, Ryan P, Lee KJ. Should multiple imputation be the method of choice for handling missing data in randomized trials? *Stat Methods Med Res.* 2018;27(9):2610-2626.

Ultrasound Guided compared to Stereotactic Navigated Ventriculoperitoneal Shunt Placement: A Randomized Controlled Study (NaVPS-Study)

Statistical analyses plan

Project number NCH-003

Analysis for Prof. Dr. med. Jehuda Soleman and Dr. med. Severina Leu

Department of Neurosurgery, University Hospital Basel

Authored by Florian Halbeisen, Surgical Outcome Research Center

Email: floriansamuel.halbeisen@usb.ch

Reviewed by Brigitta Gahl, Surgical Outcome Research Center

Email: brigitta.gahl@usb.ch

Version: 1.4 – 08.04.2025

Inhalt

1. Introduction 3
1.1. Background and rationale 3
1.2. Objectives 3
2. Study methods 3
2.1. Trial design 3
2.2. Randomisation 3
2.3. Sample size 3
2.4. Framework 3
2.5. Stratification 4
2.6. Statistical interim analyses and stopping guidance 4
3. Data management 4
3.1. Data export 4
3.2. Data validation 4
4. Statistical principles 4
4.1. General 4
4.2. Confidence intervals and p-values 4
4.3. Adherence and protocol deviations 4
4.4. Analysis populations 4
5. Trial Population 5
5.1. Screening data 5
5.2. Eligibility 5
5.3. Patient flow 5
5.4. Withdrawal/follow-up 5
5.5. Baseline patient characteristics 5
6. Analysis 5
6.1. Outcome definitions 5
6.2. Outcome derivation 6
6.3. Analysis methods 6
6.3.1 Primary analysis 6
6.4. Missing data 7
6.5. Evaluation of safety parameters 7
6.6. Statistical software 7
7. Changes from the protocol 7
7.1. Interims analysis 8
8. References 7

1. Introduction

1.1. Background and rationale

See study protocol.

1.2. Objectives

The objective of this study is to analyse the feasibility and safety of Ultrasound-guided (US-G) Ventriculoperitoneal shunt (VPS) placement. The primary objective of the study is to compare the surgical intervention of VPS placement between US-G and stereotactic navigated placement plus additional 5 minutes.

The Null-hypothesis is that there are no differences in the surgical intervention time (plus additional 5 minutes in the stereotactic navigation group) between the two methods. The Alternative Hypothesis is that US-G VPS placement has a shorter surgical intervention time compared to stereotactic navigated VPS placement.

$$H_0: Time_{\ US-G} = Time_{\ \mathrm{Stereotactic}} + 5\mathrm{min}$$

$$H_1: Time_{\ US-G} < Time_{\ \mathrm{Stereotactic}} + 5\mathrm{min}$$

2. Study methods

2.1. Trial design

This study is a prospective randomized two-arms controlled superiority trial, conducted in a primary neurosurgical centre in Switzerland.

2.2. Randomisation

Randomisation will be performed by an independent individual using a stratified simple randomisation procedure as implemented in the electronic data capture software REDCap. An allocation ratio of 1:1 will ensure a balance in sample size across both groups over time. The randomisation will be stratified according to age of the patients (under 40 years/over 40 years).

2.3. Sample size

The sample size was estimated with the aim of showing a surgical intervention time reduction of 15 minutes (min) in the intervention arm compared to the control arm. The significance level was chosen to be 5%, while the power was chosen to be (1-β) = 80%.

Based on unpublished data from a pilot study assessing the surgical intervention time in patients undergoing VPS placement using US navigation, we calculated a mean surgical intervention time of 63 min with a standard deviation (SD) of 28.65 min. Because of the additional steps involved using stereotactically navigated surgery, we assume an increased surgical invention time in those patients.

We then conservatively selected a scenario with a relatively modest difference between groups. This resulted in 58 patients with VPS placement surgery per group. Anticipating a 1% drop-out by deaths and a surgeons' non-adherence rate (change from control to treatment arm) of 5%, a total sample size of 130 patients (65 per group) was required.

2.4. Framework

All endpoints will be analysed for the superiority of US-G VPS placement compared to stereotactic navigated VPS placement.

2.5. Stratification

Unless explicitly mentioned, analyses will not be stratified.

2.6. Statistical interim analyses and stopping guidance

No interim analysis planned.

3. Data management

3.1. Data export

The entered data will be exported from the trial database (REDCap) to a statistical software package.

3.2. Data validation

Data validation and cleaning will be conducted after completion of data entry but before database lock.

4. Statistical principles

4.1. General

All recorded and derived variables will be presented by intervention group (and visits, if appropriate) using descriptive summary tables. Continuous variables will be summarised by mean and standard deviation, or median and quartiles. Categorical variables will be summarised with absolute and relative frequencies.

4.2. Confidence intervals and p-values

The statistical testing will be two-sided with a significance level of 5%. All tests will be accompanied by an effect measure with a 95% confidence interval (95% CI).

4.3. Adherence and protocol deviations

Substantial amendments are changes that affect the safety, health, rights and obligations of participants, changes in the protocol that affect study objective(s) or central research topic, changes of study site(s) or of study leader and sponsor (ClinO, Art. 29).

4.4. Analysis populations

The population for analysis will comprise all patients as randomized and with completed follow-up (no drop-outs), hence the full analysis set (FAS). All statistical analyses will be performed on the FAS according to the intention-to-treat principle (i.e. all participants will be analysed on the basis of the intervention to which they were randomly allocated).

The per-protocol (PP) set will include all participants in the FAS who fulfilled the eligibility criteria, for whom the surgery was completed as planned in the study protocol, and for whom the measurement of the primary outcome is available.

5. Trial Population

5.1. Screening data

Screening data is not collected in the eCRF but the number of screened patients will be calculated from screening logs.

5.2. Eligibility

Inclusion and exclusion criteria are defined in the study protocol. Data about reason for non-inclusion is not documented in the eCRF and will not be available.

5.3. Patient flow

A CONSORT patient flow diagram will be drawn following the CONSORT 2010 standards.[1]

5.4. Withdrawal/loss to follow-up/drop-outs

All withdrawals, drop-outs and losses to follow-up will be listed with time points and reasons (if available).

5.5. Baseline patient characteristics

Evaluations of the baseline characteristics will be based on the FAS data set. They will be presented in a descriptive summary table by intervention group. Continuous variables will be shown as mean and standard deviation or median and quartiles, categorical variables as absolute and relative frequencies. No statistical comparisons of patient characteristics at baseline will be performed.

6. Analysis

Baseline characteristics:
Preoperative patient data such as age, sex, BMI (body mass index), neurologic examination (GCS, modified Rankin Scale (mRS), Glasgow Outcome Scale (GOS), neurological symptoms (headaches, vomitus, coma, gait disturbances, dementia, urinary incontinence, motor deficit, sensory deficit, aphasia, delir, others), underlying disease causing hydrocephalus, will be collected and summarised for both groups.

6.1. Outcomes

Primary outcomes:
- Surgical intervention time («Beginn oper. Vorbereitung (Lagerung)» to «Naht neurochirurgischer Teil») in minutes, plus additional 5 minutes in the stereotactic navigation group

Secondary outcomes:
- Operation time in minutes (time in minutes from “Schnitt” to “Naht”)
- Anaesthesia time in minutes (time in minutes from “Beginn Anästhesie” to “Ende Anästhesie”)
- Number of puncture attempts
- Catheter placement (optimal vs. not optimal, grade I to IV)
- Volumetry of side ventricles pre- and postoperatively in cm^3 (number and relative change)
- Evan’s Index[2] pre- and postoperatively (number and relative change)
- Complications (infection, bleeding, complications ass. with navigation method), mortality
- Revision surgery (yes/no) and reason for revision

Outcome definitions :
The primary endpoint is the surgical intervention time. This is the time spent in the OR by the surgeon and includes the preparation time together (patient positioning, head clamping in the steretocatic navigation group) with the operation time of the neurosurgical part. Beginning ("Lagerung") and ending ("Naht neurosurgical part") of this time interval is clearly defined and will be in a standardized manner recorded by blinded anaesthesiologists. In the stereotactic navigation group, an additional 5 minutes will be added to the surgical intervention time for the preplanning of the navigation the day before the operation on the Brainlab workstation.

The optimal catheter placement has been defined as free-floating within the CSF without touching the ventricle wall or septum pellucidum, and the tip of the catheter located at the foramen of Monro showing an optimal length of the catheter. Catheters that did not fulfill all criteria have been defined as not optimally placed. Positioning of catheters will be further graded according to Yim et al. into grades I to IV (grade I: catheter terminates in the ipsilateral frontal horn, grade II: catheter terminates in contralateral frontal horn, grade III: catheter terminates in non-targeted CSF spaces, grade IV: catheter terminates intraparenchymally)[3].

6.3. Analysis methods

6.3.1 Primary analysis

Analysis of the primary endpoint will follow the intention-to-treat (ITT) principle. It will be based on the FAS with missing data of the primary outcome imputed as described in the section "Missing data" (section 6.4). Patient data will be analysed according to their intervention allocation, irrespective of actual treatment received.

The primary outcome, surgical intervention time, will be assessed using a linear regression model. The estimates will be reported with 95% confidence intervals (CI). Explanatory variables will be BMI, underlying diagnosis, experience surgeon and the use of an old burr hole.

In case the outcome variable is not normally distributed, data will be transformed or a different model, depending on the distribution of the data, chosen.

6.3.1.2. Analysis of secondary outcomes

All analysis of the secondary endpoints will follow will follow the intention-to-treat (ITT) principle. It will be based on the FAS with missing data of the primary outcome imputed as described in the section "Missing data" (section 6.4).

The continuous secondary endpoints (outcomes operation time, anaesthesia time, volumetry of side ventricles, and Evan's Index) will be assessed using linear regression models, the binary endpoints (optimal catheter placement, complications, mortality and revision surgery) will be assessed using logistic regression, and the number of puncture attempts will be assessed using a poisson regression model.

All estimates will be reported with 95% CI. IF numbers allow explanatory variables will be be BMI, underlying diagnosis, experience surgeon and the use of an old burr hole.

In case the outcome variable is not normally distributed or model assumptions are violated, data will be transformed or a different model, depending on the distribution of the data, chosen.

Deviation from the original statistical plan:
If substantial deviations of the analysis as outlined in these sections are needed for whatever reason, the protocol will be amended. All deviations of the analysis from the protocol or from the detailed analysis plan will be listed and justified in a separate section of the final statistical report.

6.4. Missing data

Missing baseline and outcome data will be summarized by study arm. As outlined above, the primary analyses will be the intent-to-treat population. In the case of missing data or drop-outs, we may adjust for further baseline variables which are associated with missing outcome data[5] or we consider multiple imputation. Both, baseline patient characteristics and outcome variables will be considered for multiple imputations. Variables with more than 50% missing values will not be used for the imputation model. Categorical variables with a frequency of 5% or less in one category will also be omitted. Continuous variables will be imputed using linear regression and binary variables using logistic regression. We will construct and analyze 20 imputed data sets and combine results using Rubin's rules.[2] Diagnostic checks, including model convergence and comparing observed versus imputed distributions, will be performed to ensure the quality of imputations.

6.5. Evaluation of safety parameters

Evaluation of safety parameters will be based on the safety population (FAS, section 4.4). They will be listed according to the treatment the patient actually received with the time points of onset. If many adverse events should be observed, we will compare frequency between treatment groups.

Safety endpoints are the following events: positioning of the catheter, number of ventricle puncture attempts, VPS dysfunction, revision rate, perioperative complications (bleeding, infection), complications due to head clamp, death, and coma.

6.6. Statistical software

The statistical analysis will be performed by Surgical Outcome Research Center using the statistical software R (Version 4.3.2, The R Foundation for Statistical Computing, Vienna, Austria).

7. Changes from the protocol

None.

8. References

1. http://www.consort-statement.org/consort-2010

2. Evans WJ. An encephalographic ratio for estimating ventricular enlargement and cerebral atrophy. Archives of Neurology & Psychiatry. 1942;47:931-937.

3. Yim B, Reid Gooch M, Dalfino JC, Adamo MA, Kenning TJ. Optimizing ventriculoperitoneal shunt placement in the treatment of idiopathic intracranial hypertension: an analysis of neuroendoscopy, frameless stereotaxy, and intraoperative CT. Neurosurg Focus. 2016;40(3):E12

4. Sullivan TR, White IR, Salter AB, Ryan P, Lee KJ. Should multiple imputation be the method of choice for handling missing data in randomized trials? Stat Methods Med Res. 2018;27(9):2610-2626.

5. Rubin DB. Multiple imputation for nonresponse in surveys. New York: John Wiley & Sons; 2004.
