# Supplementary material for: Ultrasound-guided versus stereotactically navigated ventriculoperitoneal shunt placement: a randomized clinical trial
Source: Fluids Barriers CNS. 2026 Jun 26;23:85. doi: 10.1186/s12987-026-00833-2 (PMC13309968; doi:10.1186/s12987-026-00833-2)
Supplement: Supplementary file 6 — Supplementary Material 6: Additional File 6: Additional File 6.pdf, Operation time (Linear regression) [file 12987_2026_833_MOESM6_ESM.pdf]

Additional File 6: Operation time (Linear regression)

Operation time (min)			
	**Total (N = 127)**	**Ultrasound (N = 64)**	**Stereotactic navigation (N = 63)**
**Operation time in min (Median & IQR)**	57 (46 to 72·25)	56 (46·5 to 68)	57 (46 to 74)
**Linear Regression (Operation time (min))**			
**Coefficients**	**Estimates**	**CI**	**P-value**
**Crude Model**			
**US (vs STN)**	0·7937	-7·039 - 8·626	0·841
**Adjusted Model**			
**US (vs STN)**	1·254	-6·752 - 9·26	0·747
**BMI**	-0·03731	-0·8571 - 0·7825	0·925
**Previous burr hole**	-6·786	-18·56 - 4·988	0·235
**Experience surgeon (years)**	-0·3079	-0·7369 - 0·121	0·14
**Underlying disease causing hydrocephalus**			0·04
**Subarachnoid hemorrhag**	-1·898	-14·9 - 11·11	
**Other bleeding**	-5·215	-19·83 - 9·399	
**Tumor**	3·855	-9·392 - 17·1	
**Trauma**	0·8139	-16·1 - 17·73	
**Other**	26·62	8·939 - 44·3	
