# Supplementary material for: Ultrasound-guided versus stereotactically navigated ventriculoperitoneal shunt placement: a randomized clinical trial
Source: Fluids Barriers CNS. 2026 Jun 26;23:85. doi: 10.1186/s12987-026-00833-2 (PMC13309968; doi:10.1186/s12987-026-00833-2)
Supplement: Supplementary file 7 — Supplementary Material 7: Additional File 7: Additional File 7.pdf, Operation time (min) and differences between groups [file 12987_2026_833_MOESM7_ESM.pdf]

Additional File 10: Ventricular puncture attempts (Poisson regression), multiple attempts needed (Logistic regression)

**Number of ventricular puncture attempts**			
	**Total (N = 127)**	**Ultrasound (N = 64)**	**Stereotactic navigation (N = 63)**
**Number of puncture attempts**			
**1**	117 (92·13)	55 (85·94)	62 (98·41)
**2**	8 (6·3)	7 (10·94)	1 (1·59)
**3**	2 (1·57)	2 (3·12)	0 (0)
**Poisson regression (Number of ventricular puncture attempts)**			
**Coefficients**	**Rate Ratio**	**95% CI**	**P-Value**
**Ultrasound (vs STN)**	1·15	1·048 - 1·27	0·004
**Need for multiple puncture attempts**			
	**Total (N = 127)**	**Ultrasound (N = 64)**	**Stereotactic navigation (N = 63)**
**Multiple puncture attempts**			
**Yes**	10 (7·87)	9 (14·06)	1 (1·59)
**No**	117 (92·13)	55 (85·94)	62 (98·41)
**Logistic regression**			
**Coefficients**	**Odds Ratio**	**95% CI**	**P-Value**
**Ultrasound (vs STN)**	10·15	1·822 - 190	0·005
