# Supplementary material for: Ultrasound-guided versus stereotactically navigated ventriculoperitoneal shunt placement: a randomized clinical trial
Source: Fluids Barriers CNS. 2026 Jun 26;23:85. doi: 10.1186/s12987-026-00833-2 (PMC13309968; doi:10.1186/s12987-026-00833-2)
Supplement: Supplementary file 9 — Supplementary Material 9: Additional File 9: Additional File 9.pdf, Anesthesia time (min) and differences between groups [file 12987_2026_833_MOESM9_ESM.pdf]

Additional File 8: Anaesthesia time (Linear regression)

Anaesthesia time (min)			
	**Total (N = 127)**	**Ultrasound (N = 64)**	**Stereotactic navigation (N = 63)**
**Anesthesia time in min (Median & IQR)**	176·5 (154·25 to 199·25)	168 (148 to 190)	191 (165·5 to 211·5)
**Linear regression (Anaesthesia time (min))**			
**Coefficients**	**Estimates**	**CI**	**P-value**
**Crude Model**			
**Ultrasound (vs STN)**	-18·25	-30·28 - -6·231	0·003
**Adjusted Model**			
**Ultrasound (vs STN)**	-17·7	-30·6 - -4·805	0·005
**BMI**	0·244	-1·077 - 1·565	0·703
**Previous burr hole**	-8·299	-27·27 - 10·67	0·367
**Experience surgeon (years)**	-0·01451	-0·7057 - 0·6767	0·965
**Underlying disease causing hydrocephalus**			0·904
**Subarachnoid hemorrhag**	-1·988	-22·94 - 18·96	
**Other bleeding**	10·44	-13·11 - 33·98	
**Tumor**	-3·738	-25·08 - 17·61	
**Trauma**	1·21	-26·04 - 28·46	
**Other**	0·843	-27·64 - 29·33	
