# Supplementary material for: Ultrasound-guided versus stereotactically navigated ventriculoperitoneal shunt placement: a randomized clinical trial
Source: Fluids Barriers CNS. 2026 Jun 26;23:85. doi: 10.1186/s12987-026-00833-2 (PMC13309968; doi:10.1186/s12987-026-00833-2)
Supplement: Supplementary file 11 — Supplementary Material 11: Additional File 11: Additional File 11.pdf, Catheter position (Logistic regression) [file 12987_2026_833_MOESM11_ESM.pdf]

Additional File 12: Catheter position grading (Ordinal logistic regression)

Catheter position grading			
	**Total (N = 127)**	**Ultrasound (N = 64)**	**Stereotactic navigation (N = 63)**
**Catheter position grading - 48-120h post operation**			
**Grade I**	119 (93.7)	59 (92.19)	60 (95.24)
**Grade II**	4 (3.15)	3 (4.69)	1 (1.59)
**Grade III**	4 (3.15)	2 (3.12)	2 (3.17)
**Grade IV**	0 (0)	0 (0)	0 (0)
**Catheter position grading - 2nd follow-up**			
**Grade I**	94 (74.02)	46 (71.88)	48 (76.19)
**Grade II**	2 (1.57)	2 (3.12)	0 (0)
**Grade III**	2 (1.57)	1 (1.56)	1 (1.59)
**Grade IV**	0 (0)	0 (0)	0 (0)
**NA**	29 (22.83)	15 (23.44)	14 (22.22)
**Ordinal logistic regression (Catheter position grading)**			
**Coefficients**	**Odds Ratio**	**95% CI**	**P-Value**
**48-120h post operation**			
**Ultrasound (vs STN)**	1.666	0.391 - 8.411	0.491
**2nd follow-up**			
**Ultrasound (vs STN)**	3.063	0.377 - 63.147	0.306
