# Supplementary material for: Ultrasound-guided versus stereotactically navigated ventriculoperitoneal shunt placement: a randomized clinical trial
Source: Fluids Barriers CNS. 2026 Jun 26;23:85. doi: 10.1186/s12987-026-00833-2 (PMC13309968; doi:10.1186/s12987-026-00833-2)
Supplement: Supplementary file 12 — Supplementary Material 12: Additional File 12: Additional File 12.pdf, Catheter position grading (Ordinal logistic regression) [file 12987_2026_833_MOESM12_ESM.pdf]

Additional File 14: Revision surgery (Logistic regression) and infection-related revisions

**Revision surgery**			
	**Total (N = 127)**	**Ultrasound (N = 64)**	**Stereotactic navigation (N = 63)**
**Day 0 Revision surgery**			
**Yes**	0 (0)	0 (0)	0 (0)
**No**	125 (100)	62 (100)	63 (100)
**48-120h post operation Revision surgery**			
**Yes**	2 (1·57)	2 (3·12)	0 (0)
**No**	125 (98·43)	62 (96·88)	63 (100)
**Discharge Revision surgery**			
**Yes**	0 (0)	0 (0)	0 (0)
**No**	127 (100)	64 (100)	63 (100)
**Follow-up 1 Revision surgery**			
**Yes**	1 (0·79)	1 (1·56)	0 (0)
**No**	103 (81·10)	54 (84·38)	49 (77·78)
**NA**	23 (18·11)	9 (14·06)	14 (22·22)
**Follow-up 2 Revision surgery**			
**Yes**	6 (4·72)	3 (4·69)	3 (4·76)
**No**	94 (74·02)	47 (73·44)	47 (74·6)
**NA**	27 (21·26)	14 (21·88)	13 (20·63)
**Reason for revision surgery**			
	**Ultrasound (N = 64)**	**Stereotactic navigation (N = 63)**	
**48-120h post operation - Reasons for revision surgery**			
**misplacement**	1 (50)	0 (0)	
**other**	1 (50)	0 (0)	
**Follow-up 1 - Reasons for revision surgery**			
**infection**	1 (100)	0 (0)	
**Follow-up 2 - Reasons for revision surgery**			
**distal dislocation**	0 (0)	1 (33·33)	
**infection**	0 (0)	1 (33·33)	
**infection, other**	1 (33·33)	0 (0)	
**misplacement**	1 (33·33)	0 (0)	
**other**	1 (33·33)	1 (33·33)	
**Logistic regression (revision surgery)**			
**Coefficients**	**Odds Ratio**	**95% CI**	**P-Value**
**US (vs STN) - Day 0**	-0·02	-5·24 - 5·209	0·994
**US (vs STN) - 48-120h post operation**	1·62	-0·909 - 6·56	0·228
**US (vs STN) - Discharge**	-0·02	-5·24 - 5·209	0·994
**US (vs STN) - 1st Follow-up**	1·00	-1·952 - 5·995	0·515

**US (vs STN) - 2nd Follow-up**	-0·00	-1·598 - 1·598	1
**Revision surgery due to infection**			
	**Ultrasound (N = 64)**	**Stereotactic navigation (N = 63)**	**P-Value**
**Revision surgeries due to infection**			1
**Infection**	2 (3·12)	1 (1·59)	
**No Infection**	62 (96·88)	62 (98·41)	
