# Supplementary material for: Ultrasound-guided versus stereotactically navigated ventriculoperitoneal shunt placement: a randomized clinical trial
Source: Fluids Barriers CNS. 2026 Jun 26;23:85. doi: 10.1186/s12987-026-00833-2 (PMC13309968; doi:10.1186/s12987-026-00833-2)
Supplement: Supplementary file 13 — Supplementary Material 13: Additional File 13: Additional File 13.pdf, Shunt dysfunction (Logistic regression) [file 12987_2026_833_MOESM13_ESM.pdf]

Additional File 11: Catheter position (Logistic regression)

**Catheter position (optimal vs. non-optimal)**			
	**Total (N = 127)**	**Ultrasound (N = 64)**	**Stereotactic navigation (N = 63)**
**Catheter position - 48-120h post operation**			
**optimal**	89 (70·08)	44 (68·75)	45 (71·43)
**non-optimal**	38 (29·92)	20 (31·25)	18 (28·57)
**Catheter position - 2nd follow-up**			
**optimal**	69 (70·41)	35 (71·43)	34 (69·39)
**non-optimal**	29 (29·59)	14 (28·57)	15 (30·61)
**NA**	29 (0)	15 (0)	14 (0)
**Logistic regression (Catheter position optimal vs. non-optimal)**			
**Coefficients**	**Odds Ratio**	**95% CI**	**P-Value**
**48-120h post operation**			
**Ultrasound (vs STN)**	0·88	0·4089 - 1·884	0·742
**2nd follow-up**			
**Ultrasound (vs STN)**	1·103	0·4618 - 2·647	0·825
