# Supplementary material for: Ultrasound-guided versus stereotactically navigated ventriculoperitoneal shunt placement: a randomized clinical trial
Source: Fluids Barriers CNS. 2026 Jun 26;23:85. doi: 10.1186/s12987-026-00833-2 (PMC13309968; doi:10.1186/s12987-026-00833-2)
Supplement: Supplementary file 14 — Supplementary Material 14: Additional File 14: Additional File 14.pdf, Revision surgery (Logistic regression) and infection-related revisions [file 12987_2026_833_MOESM14_ESM.pdf]

Additional File 13: Shunt dysfunction (Logistic regression)

Shunt dysfunction			
	**Total (N = 127)**	**Ultrasound (N = 64)**	**Stereotactic navigation (N = 63)**
**Shunt dysfunction - Any time**			
**Yes**	3 (2·36)	1 (1·56)	2 (3·17)
**No**	124 (97·64)	63 (98·44)	61 (96·83)
**Shunt dysfunction - 48-120h post operation**			
**Yes**	0 (0)	0 (0)	0 (0)
**No**	127 (100)	64 (100)	63 (100)
**Shunt dysfunction - Discharge**			
**Yes**	0 (0)	0 (0)	0 (0)
**No**	127 (100)	64 (100)	63 (100)
**Shunt dysfunction - 1st Follow-up**			
**Yes**	1 (0·79)	0 (0)	1 (1·59)
**No**	103 (81·1)	55 (85·94)	48 (76·19)
**Missing**	23 (18·11)	9 (14·06)	14 (22·22)
**Shunt dysfunction - 2nd Follow-up**			
**Yes**	2 (1·57)	1 (1·56)	1 (1·59)
**No**	98 (77·17)	49 (76·56)	49 (77·78)
**Missing**	27 (21·26)	14 (21·88)	13 (20·63)
**Logistic regression (Shunt dysfunction)**			
**Coefficients**	**Odds Ratio**	**95% CI**	**P-Value**
**Ultrasound (vs STN) - Any time**	0·4841	0·02217 - 5·181	0·546
