# Supplementary material for: Ultrasound-guided versus stereotactically navigated ventriculoperitoneal shunt placement: a randomized clinical trial
Source: Fluids Barriers CNS. 2026 Jun 26;23:85. doi: 10.1186/s12987-026-00833-2 (PMC13309968; doi:10.1186/s12987-026-00833-2)
Supplement: Supplementary file 15 — Supplementary Material 15: Additional File 15: Additional File 15.pdf, Complications (Logistic regression) [file 12987_2026_833_MOESM15_ESM.pdf]

Additional File 17: Ventricle volumes (Linear regression) and ventricle volumes reduction (Ordinal logistic regression)

**Ventricle Volumes (side ventricles)**			
	**Total (N = 127)**	**Ultrasound (N = 64)**	**Stereotactic navigation (N = 63)**
**48-120h post operation**			
**Absolute change (Median & IQR)**	-18 (-33 to -7)	-20·5 (-32 to -7·75)	-16 (-36·5 to -7)
**Relative change (Median & IQR)**	-13·92 (-29·71 to -6·5)	-14·53 (-25·44 to -6·53)	-11·86 (-30·51 to -6·46)
**2nd Follow-up**			
**Absolute change (Median & IQR)**	-25·5 (-54·75 to -13)	-29 (-54 to -12)	-25 (-55 to -17)
**Relative change (Median & IQR)**	-24·57 (-48·02 to -11·43)	-19·23 (-40·86 to -10·81)	-27·72 (-49·22 to -14·47)
**Linear regression (Ventricle volumes)**			
**Coefficents**	**Estimates**	**95% CI**	**P-Value**
**48-120h post operation**			
**US (vs STN) - Absolute change**	2·517	-6·92 - 11·95	0·599
**US (vs STN) - Relative change**	2·196	-7·257 - 11·65	0·646
**2nd Follow-up**			
**US (vs STN) - Absolute change**	1·918	-16·56 - 20·4	0·837
**US (vs STN) - Relative change**	1·33	-13·46 - 16·12	0·859
**Ventricle volumes reduction**			
	**Total (N = 127)**	**Ultrasound (N = 64)**	**Stereotactic navigation (N = 63)**
**48-120h post operation**			
**Ventricle volumes reduction**			
**yes**	109 (85·83)	57 (89·06)	52 (82·54)
**same**	12 (9·45)	4 (6·25)	8 (12·7)
**worse**	5 (3·94)	2 (3·12)	3 (4·76)
**NA**	1 (0·79)	1 (1·56)	0 (0)
**2nd Follow-up**			
**Ventricle volumes reduction**			
**yes**	63 (49·61)	30 (46·88)	33 (52·38)
**same**	11 (8·66)	8 (12·5)	3 (4·76)
**worse**	24 (18·9)	11 (17·19)	13 (20·63)
**NA**	29 (22·83)	15 (23·44)	14 (22·22)
**Ordinal logistic regression (Ventricle volumes reduction)**			
**Coefficients**	**Odds Ratio**	**95% CI**	**P-Value**
**48-120h post operation**			
**US (vs STN)**	0·503	0·164 - 1·417	0·205
**2nd Follow-up**			
**US (vs STN)**	1·142	0·507 - 2·591	0·749
