# Supplementary material for: Ultrasound-guided versus stereotactically navigated ventriculoperitoneal shunt placement: a randomized clinical trial
Source: Fluids Barriers CNS. 2026 Jun 26;23:85. doi: 10.1186/s12987-026-00833-2 (PMC13309968; doi:10.1186/s12987-026-00833-2)
Supplement: Supplementary file 16 — Supplementary Material 16: Additional File 16: Additional File 16.pdf, Modified Rankin Scale (Fishers exact test) [file 12987_2026_833_MOESM16_ESM.pdf]

Additional File 15: Complications (Logistic regression)

**Complications**			
	**Total (N = 127)**	**Ultrasound (N = 64)**	**Stereotactic navigation (N = 63)**
**Complications - Any time**			
**Yes**	30 (23·62)	16 (25)	14 (22·22)
**No**	97 (76·38)	48 (75)	49 (77·78)
**Complications-Intraoperative**			
**Yes**	6 (4·72)	3 (4·69)	3 (4·76)
**No**	121 (95·28)	61 (95·31)	60 (95·24)
**Complications - 48-120h post operation**			
**Yes**	8 (6·3)	6 (9·38)	2 (3·17)
**No**	119 (93·7)	58 (90·62)	61 (96·83)
**Complications - Discharge**			
**Yes**	7 (5·51)	2 (3·12)	5 (7·94)
**No**	119 (93·7)	62 (96·88)	57 (90·48)
**NA**	1 (0·79)	0 (0)	1 (1·59)
**Complications - 1st Follow-up**			
**Yes**	12 (9·45)	7 (10·94)	5 (7·94)
**No**	93 (73·23)	48 (75)	45 (71·43)
**NA**	22 (17·32)	9 (14·06)	13 (20·63)
**Complications - 2nd Follow-up**			
**Yes**	6 (4·72)	3 (4·69)	3 (4·76)
**No**	94 (74·02)	47 (73·44)	47 (74·6)
**NA**	27 (21·26)	14 (21·88)	13 (20·63)
**Logistic regression (Complications)**			
**Coefficients**	**Odds Ratio**	**95% CI**	**P-Value**
**US (vs STN) - Any time**	1·167	0·5132 - 2·676	0·712
**US (vs STN) - Intraoperative**	0·9836	0·176 - 5·496	0·984
**US (vs STN) - 48-120h post operation**	3·155	0·6949 - 22·13	0·142
**US (vs STN) - Discharge**	0·3679	0·05125 - 1·78	0·22
**US (vs STN) - 1st Follow-up**	1·312	0·3909 - 4·716	0·66
**US (vs STN) - 2nd Follow-up**	1	0·1773 - 5·641	1
