# Supplementary material for: Ultrasound-guided versus stereotactically navigated ventriculoperitoneal shunt placement: a randomized clinical trial
Source: Fluids Barriers CNS. 2026 Jun 26;23:85. doi: 10.1186/s12987-026-00833-2 (PMC13309968; doi:10.1186/s12987-026-00833-2)
Supplement: Supplementary file 17 — Supplementary Material 17: Additional File 17: Additional File 17.pdf, Ventricle volumes (Linear regression) and ventricle volumes reduction (Ordinal logistic regression) [file 12987_2026_833_MOESM17_ESM.pdf]

**Additional File 18:** Evans index (Linear regression) and Evans index improvement (Ordinal logistic regression)

| Evans Index | | | |
|---|---|---|---|
| | **Total (N = 127)** | **Ultrasound (N = 64)** | **Stereotactic navigation (N = 63)** |
| **48-120h post operation** | | | |
| **Change in Evans Index (Median & IQR)** | -0·02 (-0·04 to -0·01) | -0·02 (-0·05 to -0·01) | -0·02 (-0·03 to -0·01) |
| **Relative change in Evans Index (Median & IQR)** | -5·56 (-10 to -2·56) | -5·71 (-13·96 to -2·74) | -5·06 (-8·69 to -2·35) |
| | | | |
| **2nd Follow-up** | | | |
| **Change in Evans Index (Median & IQR)** | -0·03 (-0·05 to -0·02) | -0·03 (-0·05 to -0·02) | -0·03 (-0·07 to -0·02) |
| **Relative change in Evans Index (Median & IQR)** | -8·11 (-15·94 to -4·13) | -7·89 (-14·29 to -4·55) | -8·89 (-16·67 to -4·08) |
| **Linear regression (Evans Index)** | | | |
| **Coefficients** | **Estimates** | **95% CI** | **P-Value** |
| **48-120h post operation** | | | |
| **US (vs STN) - Absolute change** | -0·00797 | -0·0229 - 0·006957 | 0·293 |
| **US (vs STN) - Relative change** | -2·466 | -6·957 - 2·026 | 0·279 |
| **2nd Follow-up** | | | |
| **US (vs STN) - Absolute change** | -0·003878 | -0·02831 - 0·02056 | 0·753 |
| **US (vs STN) - Relative change** | -1·144 | -7·661 - 5·373 | 0·728 |
| **Evans Index improvement** | | | |
| | **Total (N = 127)** | **Ultrasound (N = 64)** | **Stereotactic navigation (N = 63)** |
| **48-120h post operation** | | | |
| **Evans Index improved** | | | |
| **improved** | 107 (84·25) | 57 (89·06) | 50 (79·37) |
| **same** | 13 (10·24) | 4 (6·25) | 9 (14·29) |
| **worse** | 5 (3·94) | 2 (3·12) | 3 (4·76) |
| **NA** | 2 (1·57) | 1 (1·56) | 1 (1·59) |
| | | | |
| **2nd Follow-up** | | | |
| **Evans Index improved** | | | |
| **improved** | 63 (49·61) | 30 (46·88) | 33 (52·38) |
| **same** | 11 (8·66) | 8 (12·5) | 3 (4·76) |
| **worse** | 24 (18·9) | 11 (17·19) | 13 (20·63) |
| **NA** | 29 (22·83) | 15 (23·44) | 14 (22·22) |
| **Ordinal logistic regression (Evans Index improvement)** | | | |
| **Coefficients** | **Odds Ratio** | **95% CI** | **P-Value** |
| **48-120h post operation** | | | |
| **US (vs STN)** | 0·446 | 0·146 - 1·233 | 0·121 |
| **2nd Follow-up** | | | |
| **US (vs STN)** | 1·142 | 0·507 - 2·591 | 0·748 |
