# Supplementary material for: Ultrasound-guided versus stereotactically navigated ventriculoperitoneal shunt placement: a randomized clinical trial
Source: Fluids Barriers CNS. 2026 Jun 26;23:85. doi: 10.1186/s12987-026-00833-2 (PMC13309968; doi:10.1186/s12987-026-00833-2)
Supplement: Supplementary file 18 — Supplementary Material 18: Additional File 18: Additional File 18.pdf, Evans index (Linear regression) and Evans index improvement (Ordinal logistic regression) [file 12987_2026_833_MOESM18_ESM.pdf]

Additional File 16: Modified Rankin Scale (Fishers exact test)

**Modified Ranking scale**			
	**Total (N = 127)**	**Ultrasound (N = 64)**	**Stereotactic navigation (N = 63)**
**Modified Ranking Scale - 48-120h post operation**			
**p-value***	**0·227**		
**Favourable outcome (mRS 0 - 2)**	64 (50·39)	36 (56·25)	28 (44·44)
**Bad outcome (mRS 3 - 5)**	63 (49·61)	28 (43·75)	35 (55·56)
**Dead (mRS 6)**	0 (0)	0 (0)	0 (0)
**Modified Ranking Scale - Discharge**			
**p-value***	**0·197**		
**Favourable outcome (mRS 0 - 2)**	66 (51·97)	37 (57·81)	29 (46·03)
**Bad outcome (mRS 3 - 5)**	59 (46·46)	27 (42·19)	32 (50·79)
**Dead (mRS 6)**	2 (1·57)	0 (0)	2 (3·17)
**Modified Ranking Scale - Follow-up 1**			
**p-value***	**0·345**		
**Favourable outcome (mRS 0 - 2)**	68 (64·76)	38 (69·09)	30 (60)
**Bad outcome (mRS 3 - 5)**	36 (34·29)	17 (30·91)	19 (38)
**Dead (mRS 6)**	1 (0·95)	0 (0)	1 (2)
**Modified Ranking Scale - Follow-up 2**	**0·029**		
**p-value***			
**Favourable outcome (mRS 0 - 2)**	68 (68·69)	39 (79·59)	29 (58)
**Bad outcome (mRS 3 - 5)**	31 (31·31)	10 (20·41)	21 (42)
***Fishers exact test**			
